# Contrasting associations of blood lipids with risk of myocardial infarction in Chinese and European adults

**DOI:** 10.1093/ehjopen/oeaf119

**Published:** 2025-09-19

**Authors:** Hanyu Wang, Robert Clarke, Christiana Kartsonaki, Iona Millwood, Robin Walters, Michael Hill, Daniel Avery, Canqing Yu, DianJian-Yi Sun, Jun Lv, Shanpeng Li, Liming Li, Zhengming Chen, Neil Wright, Derrick A Bennett

**Affiliations:** Clinical Trial Service Unit, Nuffield Department of Population Health, University of Oxford, Big Data Institute Building, Roosevelt Drive, Oxford OX3 7LF, UK; Clinical Trial Service Unit, Nuffield Department of Population Health, University of Oxford, Big Data Institute Building, Roosevelt Drive, Oxford OX3 7LF, UK; Clinical Trial Service Unit, Nuffield Department of Population Health, University of Oxford, Big Data Institute Building, Roosevelt Drive, Oxford OX3 7LF, UK; Clinical Trial Service Unit, Nuffield Department of Population Health, University of Oxford, Big Data Institute Building, Roosevelt Drive, Oxford OX3 7LF, UK; Clinical Trial Service Unit, Nuffield Department of Population Health, University of Oxford, Big Data Institute Building, Roosevelt Drive, Oxford OX3 7LF, UK; Clinical Trial Service Unit, Nuffield Department of Population Health, University of Oxford, Big Data Institute Building, Roosevelt Drive, Oxford OX3 7LF, UK; Clinical Trial Service Unit, Nuffield Department of Population Health, University of Oxford, Big Data Institute Building, Roosevelt Drive, Oxford OX3 7LF, UK; Department of Epidemiology and Biostatistics, School of Public Health, Peking University Health Science Centre, Beijing 100191, China; Peking University Center for Public Health and Epidemic Preparedness and Response, Beijing 100191, China; Key Laboratory of Epidemiology of Major Diseases, Ministry of Education, Beijing 100191, China; Department of Epidemiology and Biostatistics, School of Public Health, Peking University Health Science Centre, Beijing 100191, China; Peking University Center for Public Health and Epidemic Preparedness and Response, Beijing 100191, China; Key Laboratory of Epidemiology of Major Diseases, Ministry of Education, Beijing 100191, China; Department of Epidemiology and Biostatistics, School of Public Health, Peking University Health Science Centre, Beijing 100191, China; Peking University Center for Public Health and Epidemic Preparedness and Response, Beijing 100191, China; Key Laboratory of Epidemiology of Major Diseases, Ministry of Education, Beijing 100191, China; Qingdao Centre for Disease Control, Qindao, Shandong 266033, China; Department of Epidemiology and Biostatistics, School of Public Health, Peking University Health Science Centre, Beijing 100191, China; Peking University Center for Public Health and Epidemic Preparedness and Response, Beijing 100191, China; Key Laboratory of Epidemiology of Major Diseases, Ministry of Education, Beijing 100191, China; Clinical Trial Service Unit, Nuffield Department of Population Health, University of Oxford, Big Data Institute Building, Roosevelt Drive, Oxford OX3 7LF, UK; Clinical Trial Service Unit, Nuffield Department of Population Health, University of Oxford, Big Data Institute Building, Roosevelt Drive, Oxford OX3 7LF, UK; Clinical Trial Service Unit, Nuffield Department of Population Health, University of Oxford, Big Data Institute Building, Roosevelt Drive, Oxford OX3 7LF, UK

**Keywords:** Blood lipids, Myocardial infarction, Chinese and European populations

## Abstract

**Aims:**

Little is known about the importance of blood lipids for risk of myocardial infarction (MI) in Chinese vs. European populations. We compared the associations with MI of apolioprotein B (ApoB) vs. low-density lipoprotein cholesterol (LDL-C) and remnant-cholesterol (remnant-C) vs. triglycerides in the China Kadoorie Biobank (CKB) and UK Biobank (UKB).

**Methods and results:**

Plasma levels of LDL-C, high-density lipoprotein-cholesterol (HDL-C), apolipoprotein B (ApoB), apolipoprotein A1 (ApoA1), non-HDL-C, remnant-C, LDL-C/ApoB, and HDL-C/ApoA1 ratios were measured in a nested case-control study of MI (948 cases, 6101 controls) in CKB and a prospective study (5344 cases in 279 989 participants) in UKB. Associations of lipids with MI were assessed using logistic regression in CKB and Cox regression in UKB after adjustment for confounders and correction for regression dilution. The mean levels of LDL-C were about 30% lower in CKB than in UKB [2.3 (0.6) vs. 3.7 (0.8) mmol/L], but mean levels of HDL-C were comparable [1.3 (0.3) vs. 1.5 (0.4) mmol/L], as were those for triglycerides [1.8 (1.1) vs. 1.7 (1.1) mmol/L]. While the rate ratios (RRs) of MI for 1 SD higher usual levels of LDL-C in Chinese were about half those in Europeans (1.27; 1.13–1.44 vs. 1.55; 1.49–1.61), the corresponding RRs for ApoB or non-HDL with MI were comparable between Chinese and Europeans.

**Conclusion:**

The findings reinforce current guidelines for primary prevention of atherosclerotic cardiovascular disease (ASCVD) in China that advocate initiation of statin treatment in individuals at high-risk of ASCVD rather than high levels of LDL-C.

## Graphical Abstract

**Graphical Abstract:**
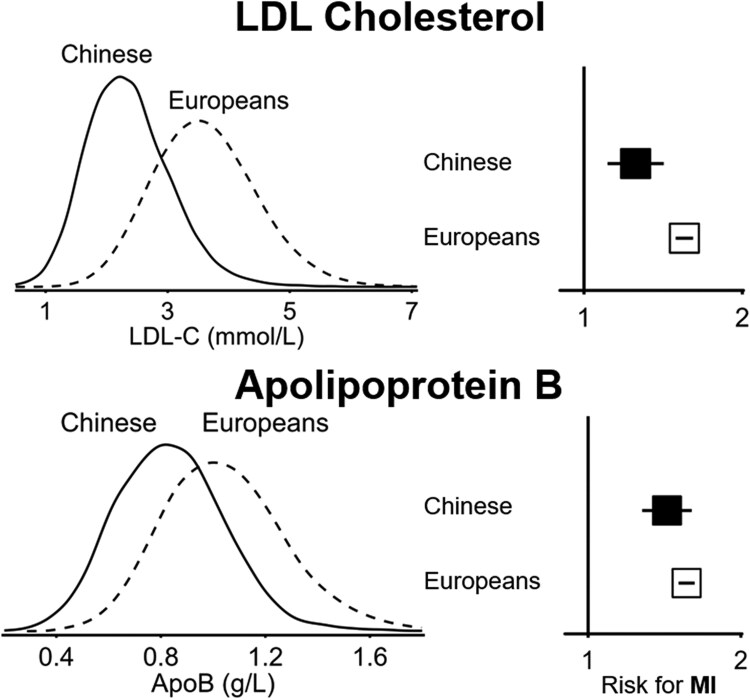

## Introduction

Mean levels of low-density lipoprotein cholesterol (LDL-C) are lower in Chinese than in Europeans in population studies of statin-free individuals, but whether such differences in distribution influence the shape and strength of associations of cholesterol fractions, apolipoproteins, remnant cholesterol (remnant-C) or lipid ratios with risk of myocardial infarction (MI) between Chinese and European ancestry populations is uncertain.^1^

Higher plasma levels of LDL-C are linearly and positively associated with higher risks of MI and higher levels of high-density lipoprotein cholesterol (HDL-C) are inversely associated with MI in European populations.^2^ Plasma levels of apolipoprotein B (ApoB) are highly correlated with LDL-C and apolipoprotein A1 (ApoA) levels are highly correlated with high-density lipoprotein cholesterol (HDL-C). Since each of the atherogenic lipoprotein particles contains a single molecule of apolioprotein B (ApoB), plasma levels of ApoB provide an indirect measure of the number of atherogenic particles. Previous studies in European populations reported stronger associations of ApoB (i.e, number of ApoB particles) with MI than LDL-C and perhaps reflecting a greater effect on risk of MI of the number of atherogenic particles than their cholesterol content.^3–14^

The Emerging Risk Factors Collaboration (ERFC) meta-analysis of prospective observational studies reported that measurement of additional lipids [apolipoprotein B (ApoB) or non-HDL-C (i.e. total cholesterol minus HDL-C])] yielded only modest improvement for risk prediction beyond conventional lipids (LDL-C, HDL-C and triglycerides).^2^ Moreover, the ERFC advocated limiting lipid measures for risk prediction of vascular disease to total cholesterol and HDL-C or apolipoproteins without any requirement for fasting or including triglyceride measurements.^2^ In contrast, other studies reported that ApoB is more strongly associated with MI than LDL-C,^4–14^ but no previous studies compared the associations of LDL-C and ApoB with risk of MI in statin-free participants in both Chinese and European populations that differ in their diet and lifestyle.

Beyond ApoB and LDL-C, Nordestgaard and colleagues advocated measurement of remnant-cholesterol (remnant-C), defined as the remaining lipids after subtraction of LDL-C and HDL-C from total cholesterol, as an additional lipid target for prediction of MI beyond LDL-C levels.^15^ Remnant-C are triglyceride-rich lipoproteins, including very low-density lipoproteins, intermediate-density lipoproteins, and chylomicrons. Other studies reported that lipid ratios, including LDL-C/ApoB (reflecting LDL particle size), and HDL-C/ApoA1 (reflecting HDL particle size) may be independently related to risk of MI beyond LDL-C alone.^3,16^ While previous small studies had suggested that the associations of cholesterol fractions with MI yielded comparable results between Chinese and Europeans,^17–19^ little is known about the relative importance for risk of MI of other lipid measures beyond conventional lipid assays in these populations.^1,20^

The aims of the present study were to compare the shape and strength of associations with MI for equivalent proportional differences in (i) non-HDL-C or ApoB vs. LDL-C; (ii) remnant-C vs. triglycerides; and (iii) ApoA1 vs. HDL-C; and (iv) LDL-C/ApoB and HDL-C/ApoA1 ratios in Chinese and European populations before and after additional adjustment for other lipid measurements.

## Methods

### Study populations

This study compared the associations of plasma lipids and risk of MI using stored blood samples collected at enrolment for Chinese adults in the China Kadoorie Biobank (CKB) and Europeans in UK Biobank, respectively. The analyses involved a subset of participants in a nested case–control study of MI cases and controls in CKB and statin-free participants in UKB.^21,22^ The CKB study recruited 512 724 adults from 10 diverse areas (5 urban and 5 rural) in China between 2004 and 2008. Data were collected at baseline on socio-demographic and lifestyle factors, medical history and use of medication using an interview-administered questionnaire (see Supplementary material online, ***Table S1***). Physical measurements (height, weight, blood pressure and lung function) were also recorded. Study participants provided a 10 mL non-fasting blood sample in vacutainers containing EDTA (with time since last meal recorded) and plasma aliquots were stored in liquid nitrogen. Repeat surveys conducted in 2008 and 2013–14 in a random subset of about 5% of study participants collected identical information as at baseline (with some enhancements) and provided repeat lipid measures to correct for regression dilution bias.^23^ Follow-up was obtained by electronic linkage to local death and disease registries and all hospitalizations via health insurance records using a unique personal identification number.^22^ The present nested case–control study of MI in CKB included 948 MI cases and 6101 controls in which all non-fatal or fatal MI cases (ICD-10 codes I21 – I23) were selected from among those with no prior history of MI or stroke and no statin use at baseline.^20,24^ Details of the procedures for selection of MI cases are outlined in Supplementary material online, ***Figure S1***. Ethics approval for CKB was provided by University of Oxford and appropriate Chinese authorities and all participants provided written informed consent.^22^

The UKB study recruited 502 504 individuals from 22 assessment centres throughout the UK for a prospective study and participants completed a baseline survey between 2006 and 2010.^21^ Participants provided information on their socio-demographic and lifestyle factors, medical history, and use of medication using a touch screen self-administered questionnaire. Physical measurements included height, weight, blood pressure and lung function. Repeat surveys of random subsets were conducted between 2012 and 2021 that included repeat measures of blood lipids to correct for regression dilution bias.^23^ Incident non-fatal or fatal MI events occurring during follow-up were obtained by electronic linkage to national death registries and hospital episode statistics using a unique personal identification number. Participants with a history of MI or stroke (*n* = 22 770) and use of statin therapy at baseline (*n* = 84 632) were excluded and the analyses were restricted to white British participants (excluding *n* = 59 918 non-White participants). After excluding participants with missing lipid values (*n* = 50 154), a total of 279 989, participants were included in the present analyses. Details of the inclusion criteria for the CKB and UKB participants are provided in Supplementary material online, *Figure S1* and Supplementary material online, *Table S2*. Ethics approval was provided by the University of Oxford Research Ethics Committee and all participants provided written informed consent.

### Laboratory methods

In CKB, plasma lipids and apolipoprotein measurements were conducted at the Wolfson Laboratory, Nuffield Department of Population Health, University of Oxford, UK. LDL-C and HDL-C were measured using N-geneous reagents, and other lipids and apolipoproteins were measured using immunoturbidimetric assays on Beckman Coulter AU680 clinical chemistry analysers.^20^ In UKB, cholesterol was measured using CHO-POD analysis, while apolipoproteins and triglycerides were assessed using immunoturbidimetric assays on a Beckman Coulter AU5800 clinical chemistry analyser at the University of Manchester, UK.^25^ Both laboratories used identical assays to measure lipids in CKB and UKB, and both were accredited with the International Organization for Standardization (ISO) 17025 to provide consistent testing and calibration, which should minimise any biases due to differences in lipid assays and laboratory procedures.

### Statistical methods

Baseline characteristics are presented as mean (SD) values or numbers and percentages. Spearman correlation coefficients were used to assess associations of individual lipids with each other after adjustment for age and sex. Plasma levels of triglycerides and remnant-C were log-transformed prior to analysis so that values were approximately normally distributed. Rate ratios (RRs) were estimated using logistic regression in CKB and Cox regression in UKB, given both odds ratios and hazard ratios approximate RRs when the outcome is relatively rare.^26^ In CKB, RRs of MI were estimated for fifths of lipid traits, adjusting for age, sex, region, BMI, diabetes, alcohol use, smoking status, physical activity, education, SBP, antihypertensive medication and ambient temperature (and its square). In UKB, RRs of MI were estimated using Cox regression with time in study as timescale, stratified by 5-year age-at-risk groups and sex, and adjusted for BMI, diabetes, alcohol use, smoking status, physical activity, ethnicity, Townsend Deprivation Index, years of education and antihypertensive medication with 95% CIs estimated using the variance of the log risk.^27^ Additional analyses were conducted with sequential adjustment for all relevant confounding factors. The likelihood ratio (LR) tests with their associated χ^2^ values were computed after sequential adjustment for potential confounding factors to assess the magnitude of confounding. In addition, associations of selected apolioproteins and cholesterol fractions were mutually adjusted for each other using Ridge regression models to explore the independence of LDL-C vs. ApoB and HDL-C vs. ApoA1.^28^ Repeat measures of all lipid measures (except for lipid ratios) were used to correct for regression dilution bias using the MacMahon-Peto method (see Supplementary material online, ***Table S3***).^23^ Sensitivity analyses excluded participants with prevalent diabetes in CKB and explored associations at low LDL-C levels in UKB to facilitate comparisons with CKB. Additional sensitivity analyses compared the effects on MI risk for differences in LDL-C or ApoB in CKB when estimated using Cox proportional hazards or logistic regression models. Further sensitivity analyses examined the associations of LDL-C and ApoB with MI after stratification by plasma triglyceride levels. The CKB data release 18.01 was used. All analyses were conducted in R version 4.2.2.

## Results

*Table 1* compares the baseline characteristics separately for MI cases and controls in CKB and for all participants in UKB. Both populations had similar mean age, but UKB included a higher proportion of females than CKB and a higher proportion of participants with higher levels of full-time education. Among lifestyle factors, a higher percentage of Chinese (CKB controls) than European men were current smokers (61.2% vs. 11.9%), but a higher proportion of European than Chinese men were current drinkers (80.6% vs. 28.2%). Few Chinese women were current smokers or current drinkers. Both populations had similar levels of physical activity. A higher proportion of Chinese than Europeans had diabetes at baseline (4.8% vs. 1.6%). Use of antihypertensive medication was similar in CKB and UKB (11.5% vs. 12.8%). Mean levels of BMI in CKB participants were lower than in UKB (23.0 kg/m² vs. 27.0 kg/m²), but mean levels of SBP were comparable in CKB and UKB (135.0 mmHg vs. 137.0 mmHg).

**Table 1:** Baseline characteristics of participants in China Kadoorie Biobank and UK Biobank studies

Characteristics Mean (SD) or %	CKB		UKB (*n* = 279 989)
	MI cases (*n* = 948)	Controls (*n* = 6101)	
**Demographics**			
Age, years	52.4 (7.1)	58.3 (10.9)	55.5 (8.0)
Female	39.6	47.9	57.1
Educated to middle school or above^a^	46.1	32.8	62.0
**Lifestyle factors**			
Male current smoker	75.1	61.2	11.9
Female current smoker	3.5	3.2	8.5
Male weekly drinker	27.0	28.2	80.6
Female weekly drinker	1.3	1.9	66.3
Total physical activity, MET-h/day^b^	5.5 (4.4)	6.5 (4.5)	6.4 (6.5)
**Medical history and medications**			
Diabetes^c^	11.9	4.8	1.6
Hypertension^d^	54.2	41.5	49.7
Antihypertensive use	17.7	11.5	12.8
**Physical measurements**			
BMI, kg/m²	24.0 (3.6)	23.0 (3.3)	27.0 (4.6)
SBP, mmHg	140.8 (25.7)	135.0 (21.3)	137.0 (18.5)
DBP, mmHg	82.7 (13.6)	77.3 (11.0)	82.3 (10.1)
**Lipid measurements**			
Total cholesterol, mmol/L	4.8 (1.0)	4.6 (0.9)	5.9 (1.0)
Apolipoprotein B, g/L	0.9 (0.2)	0.8 (0.2)	1.1 (0.2)
Non-HDL-C, mmol/L	3.6 (1.1)	3.3 (0.9)	4.4 (1.0)
LDL-C, mmol/L	2.4 (0.8)	2.3 (0.6)	3.7 (0.8)
LDL-C/apolipoprotein B, mmol/g	2.7 (0.3)	2.8 (0.3)	3.5 (0.2)
Triglycerides, mmol/L	2.0 (1.3)	1.8 (1.1)	1.7 (1.0)
Remnant cholesterol, mmol/L	1.2 (0.5)	1.0 (0.4)	0.7 (0.3)
Apolipoprotein A1, g/L	1.2 (0.2)	1.4 (0.2)	1.6 (0.3)
HDL-C, mmol/L	1.2 (0.3)	1.3 (0.3)	1.5 (0.4)
HDL-C/apolipoprotein A1, mmol/g	0.9 (0.1)	0.9 (0.1)	0.9 (0.1)

^a^In UKB, this category includes those who were educated to A levels/AS levels/equivalent and above.

^b^Total physical activity excludes work-related activity. UKB has around 20% missing data in self-reported physical activity.

^c^In UKB, baseline diabetes includes self-reported cases or those participants with a HbA1c level above 48 mmol/mol; In CKB, it includes self-reported cases and screen-detected cases (a blood glucose level ≥7.0 mmol/L and a fasting time >8 h, a blood glucose level ≥11.1 mmol/L and a fasting time <8 h, or a fasting blood glucose level ≥7.0 mmol/L).

^d^In both UKB and CKB, prevalent hypertension was defined as self-reported hypertension, or having SBP ≥ 140 mmHg or DBP ≥ 90 mmHg, or currently taking antihypertensive medications.

Mean levels of LDL-C level were about 30% lower in CKB than in UKB (2.3 mmol/L vs. 3.7 mmol/L), as were mean levels of non-HDL-C (3.3 mmol/L vs. 4.4 mmol/L). Age- and sex-adjusted correlations of lipid traits are shown in Supplementary material online, *Figure S2*. In both populations, LDL-related measures were positively correlated with triglyceride-related measures, while HDL-related measures were inversely correlated with triglyceride-related measures. In UKB, the prevalence of diabetes was inversely associated with fifths of LDL-C/ApoB and HDL-C/ApoA1 ratios (see Supplementary material online, *Table S4*). In CKB, the prevalence of diabetes was similar across fifths of LDL-C/ApoB, but was positively associated with fifths of remnant-C, and inversely associated with fifths of HDL-C/ApoA1.

### LDL-C-related lipid traits

**Figure 1:**
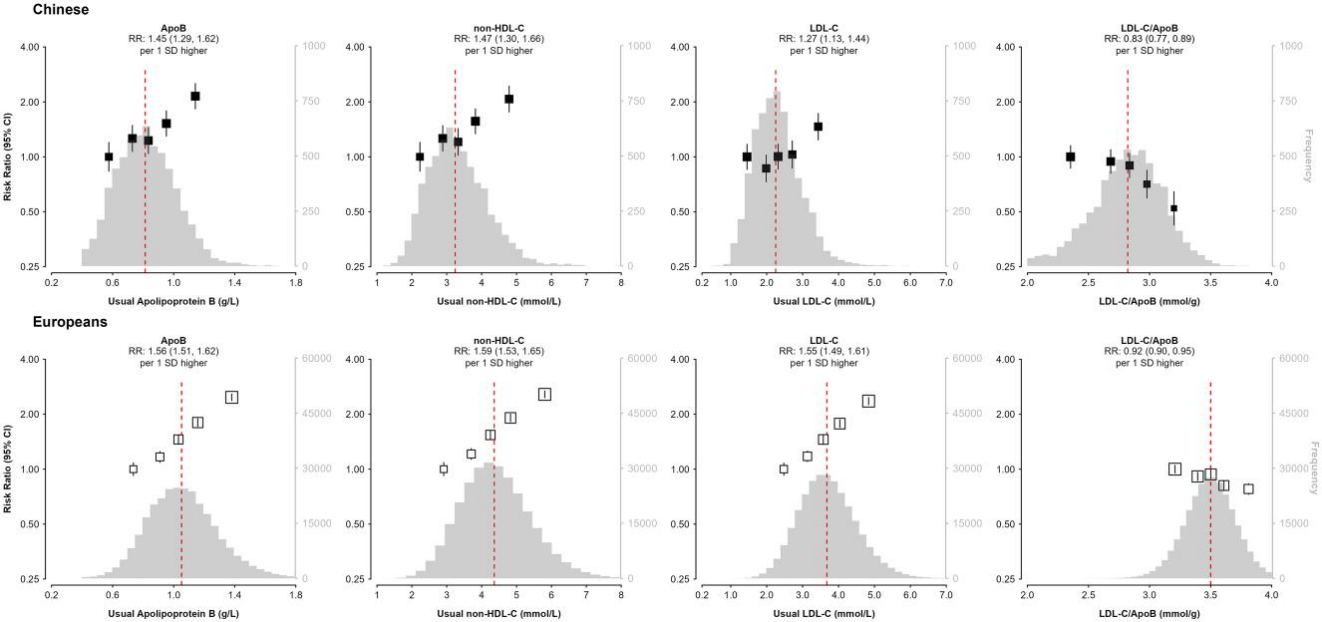
Adjusted risk ratios (95% CI) of myocardial infarction for low-density lipoprotein–related measures in Chinese and Europeans. In the Chinese population, the rate ratios were adjusted for age, sex, region, body mass index, diabetes, alcohol use, smoking status, systolic blood pressure, physical activity, educational attainment, antihypertensive medications, mean temperature, and mean temperature-squared. In European populations, the risk ratios were stratified by 5-year age-at-risk bands and sex and adjusted for body mass index, diabetes, alcohol use, smoking status, systolic blood pressure, physical activity, Townsend Deprivation Index, years of education, and antihypertensive medication. The distributions of blood lipids in the Chinese population were obtained from baseline samples in controls only. The red dashed lines indicate median values. SDs are from baseline measurements and can be referred from *Table 1*. ApoB, apolipoprotein B; LDL-C, low-density lipoprotein cholesterol; non-HDL-C, non-high-density lipoprotein cholesterol; RR, risk ratio.

*Figure 1* shows that the distributions of plasma levels of all LDL-related biomarkers were about 1 SD lower in Chinese than in Europeans (and the range of LDL-C values varied from 1 to 6 mmol/L). The shape of the association of usual plasma levels of LDL-C with MI was non-linear in Chinese adults, with a weaker strength of association with MI at LDL-C levels below 3 mmol/L (*Figure 1*). The non-linearity persisted after excluding individuals with diabetes at baseline in CKB (see Supplementary material online, *Figure S3*). Below 3 mmol/L, there was also no evidence of any association of usual plasma levels of LDL-C with MI in European adults (see Supplementary material online, *Figure S4*). Overall, the RR (95% CI) of 1 SD higher usual plasma levels of LDL-C with MI was only half of that in Chinese than in European adults (1.27; 1.13–1.44 vs. 1.55; 1.49–1.61 per 1 SD higher levels, respectively). In contrast with LDL-C, both non-HDL-C and ApoB and all other LDL-related lipid markers were equally strongly associated with MI in Chinese and European adults. The χ^2^ values for ApoB were greater than for LDL-C in Chinese adults (41.1 vs. 17.3), but the differences were less extreme in European adults (563.7 vs. 496.2: Supplementary material online, *Figure S5*).

### Triglyceride-related lipid traits

**Figure 2:**
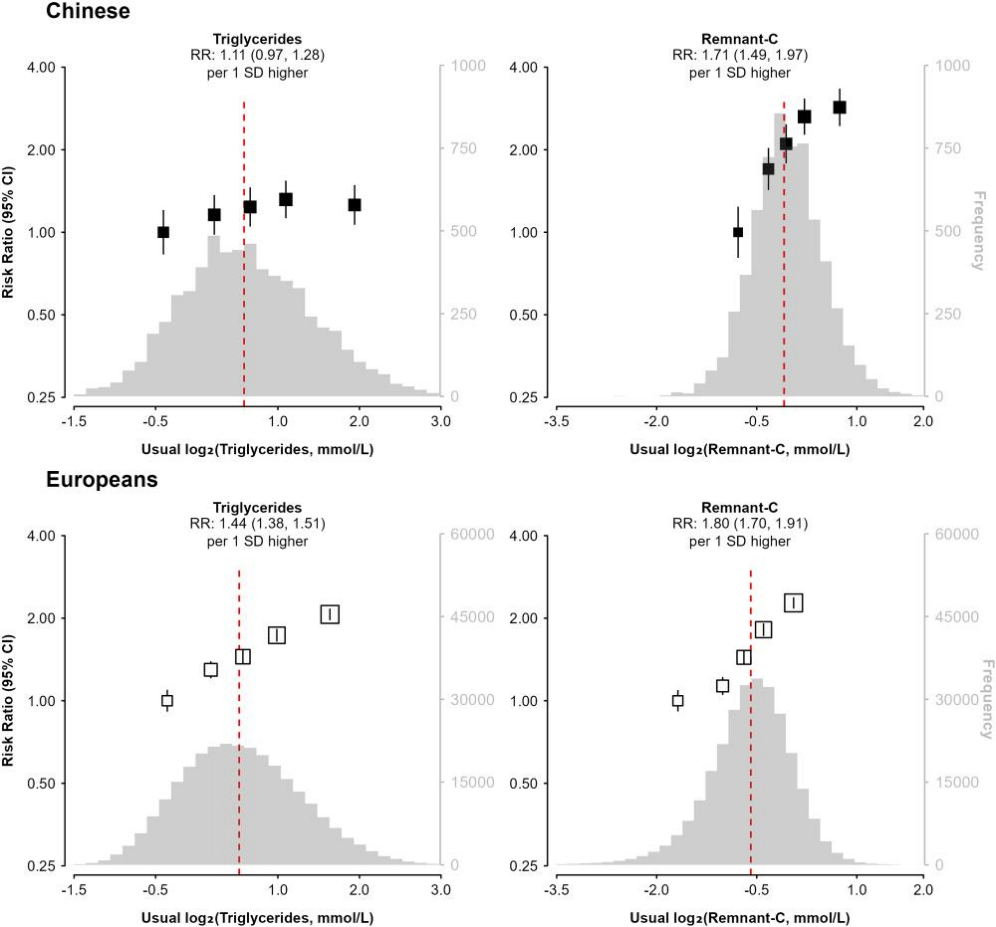
Adjusted risk ratios (95% CI) of myocardial infarction for triglyceride-related measures in Chinese and Europeans. In the Chinese population, the risk ratios were adjusted for age, sex, region, body mass index, diabetes, alcohol use, smoking status, systolic blood pressure, physical activity, educational attainment, antihypertensive medications, mean temperature, and mean temperature-squared. In European populations, the risk ratios were stratified by 5-year age-at-risk bands and sex, and adjusted for body mass index, diabetes, alcohol use, smoking status, systolic blood pressure, physical activity, Townsend Deprivation Index, years of education, and antihypertensive medication. The distributions of blood lipid levels in the China Kadoorie Biobank were obtained from baseline samples in controls only. The red dashed lines indicate median values. SDs are from baseline measurements and can be referred from *Table 1*. RR, risk ratio; Remnant-C, remnant cholesterol.

Higher usual plasma levels of triglycerides were associated with higher RRs (95% CI) of MI in both populations (*Figure 2*), but the RR were substantially weaker in Chinese than in European adults (1.11, 0.97–1.28 vs. 1.44, 1.38–1.51 per 1 SD higher levels, respectively), despite having broadly similar mean levels. Higher usual plasma levels of remnant-C had similar RRs (95% CI) for 1 SD higher levels with MI risk in Chinese and in European adults (1.71; 1.49–1.97 vs. 1.80; 1.70–1.91, respectively). In both populations, higher usual plasma levels of remnant-C were more strongly associated with MI than equivalent differences in triglycerides, with a 17-fold difference in the χ^2^ values in the Chinese population (χ^2^ of 71.1 vs. 4.2 for remnant-C vs. triglycerides, respectively) and about 2-fold greater difference in European populations (χ^2^ of 411.4 vs. 237.9 for remnant-C vs. triglycerides after adjustment for confounding factors: Supplementary material online, *Figure S5*). After additional adjustment for ApoB and ApoA1, the associations of usual plasma levels of triglycerides with MI were no longer significant in Chinese and greatly attenuated in European populations (see Supplementary material online, *Figure S5*). Higher usual levels of plasma levels of remnant-C remained associated with MI in both populations, although the RRs were attenuated after adjustment for the other lipids.

### Effect of adjustment for confounding factors on associations of lipids with MI

**Figure 3:**
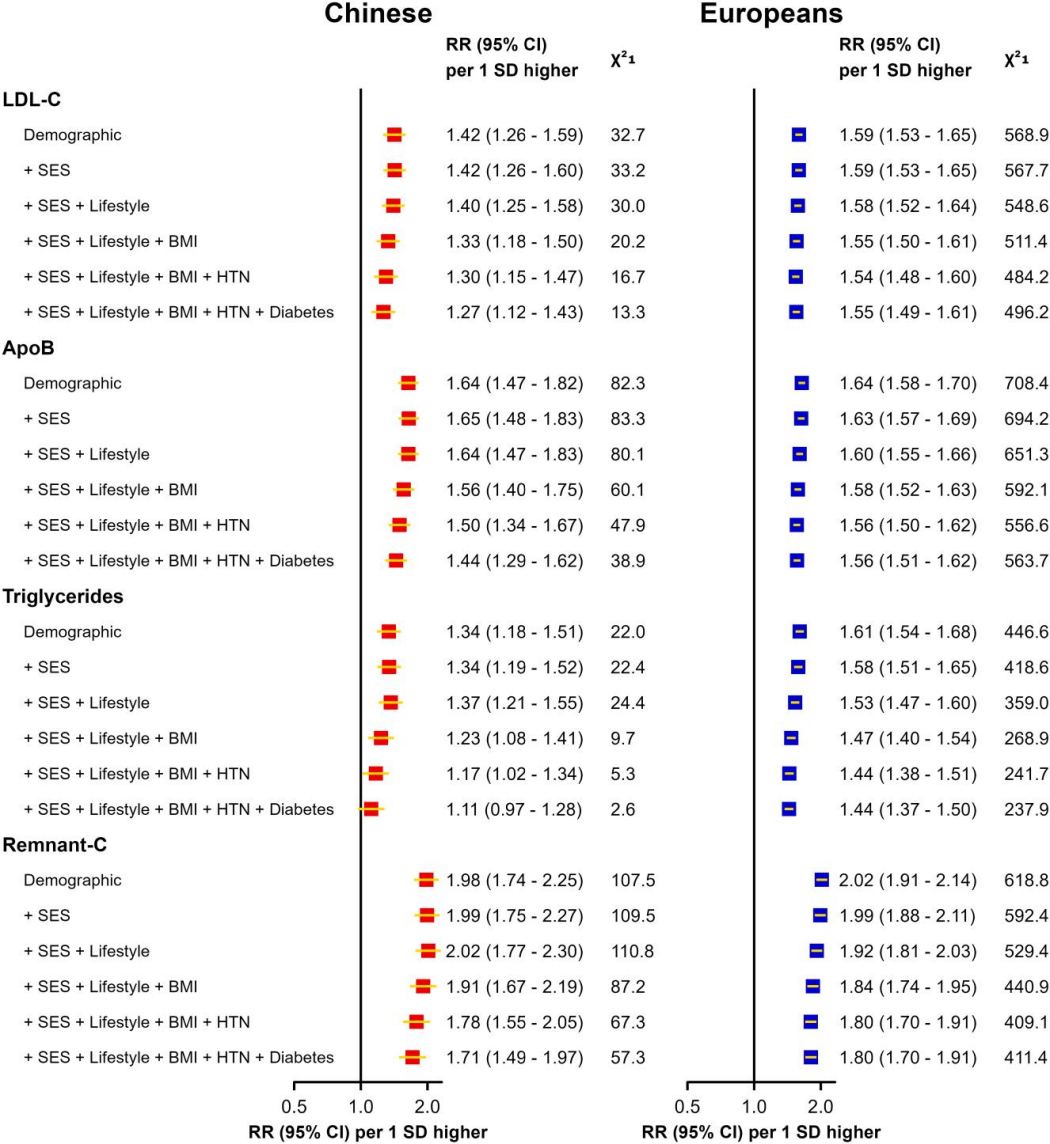
Risk ratios (95%CI) of myocardial infarction for low-density lipoprotein–related and triglyceride-related measures with sequential adjustment for confounders. In the Chinese population, the demographic model included age, sex, and region. Socioeconomic status included education, and lifestyle factors included alcohol use, smoking, and physical activity. Blood pressure refers to systolic blood pressure and antihypertensive medication use. In the European population, the demographic model was stratified by age and sex. SES included the Townsend deprivation index and education, while lifestyle factors include alcohol use, smoking, and physical activity. Blood pressure refers to systolic blood pressure and antihypertensive medication use.

*Figure 3* assesses the magnitude of effect of adjustment for confounding factors on the associations of LDL-C, ApoB, triglycerides and remnant-C with MI in both populations. Comparison of the changes in chi-squared statistics for associations with MI after sequential incremental adjustment for individual confounders in *Figure 3* suggests that the associations of LDL-C with MI were more susceptible to confounding factors (chiefly, BMI, hypertension, and diabetes) compared with ApoB and these effects were greater in Chinese than in Europeans. Likewise, the associations of triglycerides with MI were more susceptible to confounders than remnant-C in both populations, and the effects of confounding of triglycerides were also greater in Chinese than in European populations.

### HDL-C-related lipid traits

Higher usual plasma levels of ApoA1 were inversely associated with risk of MI in both populations (see Supplementary material online, *Figure S6*), but the RRs (95% CI) per 1 SD higher levels were more extreme in Chinese than in European populations (0.45; 0.39–0.52 vs. 0.72; 0.69–0.75, respectively). The strength of associations of usual plasma levels of HDL-C with MI were similar to those for usual plasma levels of ApoA1 in European populations, but there was an attenuation in the strength of the association of HDL-C with MI when HDL-C levels were greater than 1 mmol/L in the Chinese population (see Supplementary material online, *Figure S6*).

### Lipid ratios

**Figure 4:**
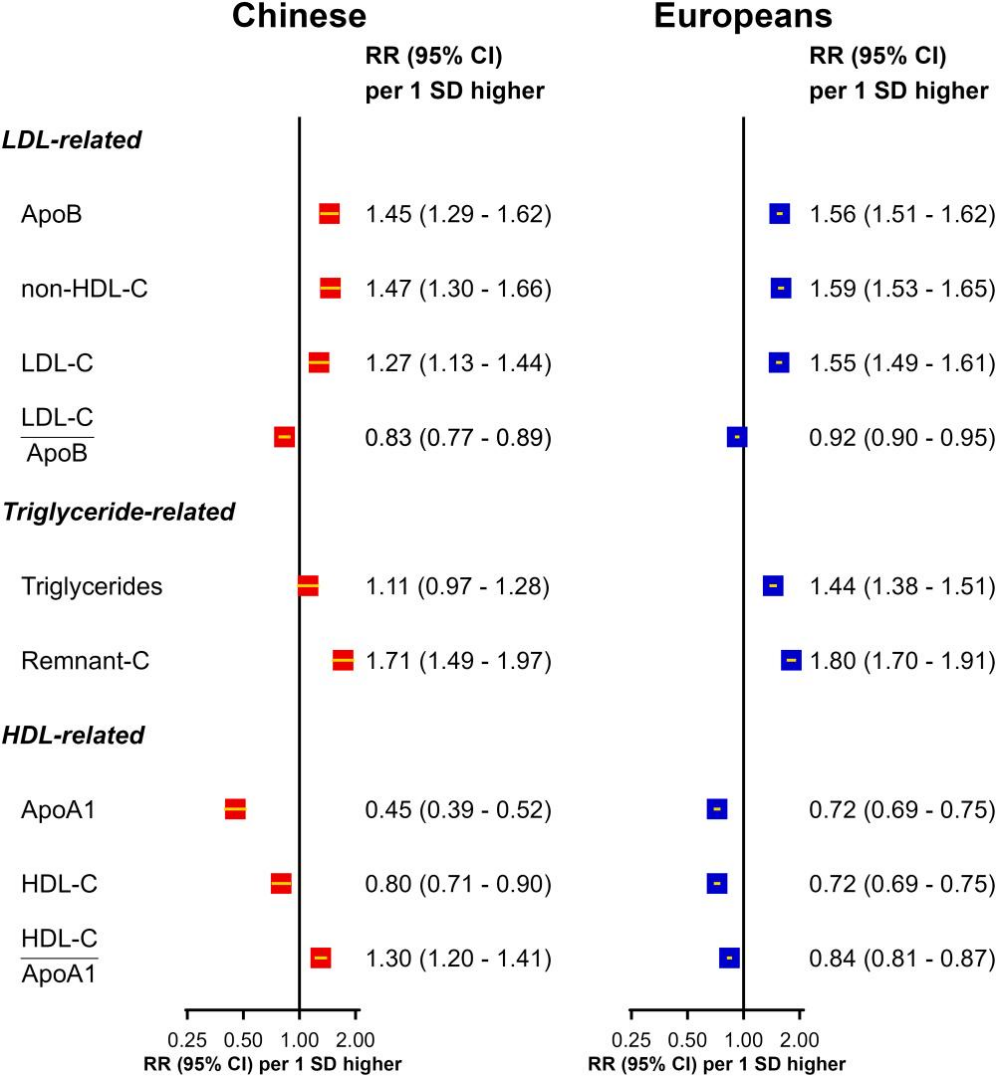
Adjusted risk ratios (95% CI) of myocardial infarction in Chinese and Europeans. In the Chinese population, the risk ratios adjusted for age, sex, region, body mass index, diabetes, alcohol use, smoking status, systolic blood pressure, physical activity, educational attainment, antihypertensive medications, mean temperature, and mean temperature-squared. In European populations, the risk ratios were stratified by 5-year age-at-risk groups and sex, with adjustments for body mass index, diabetes, alcohol use, smoking status, SBP, physical activity, Townsend Deprivation Index, years of education, and antihypertensive medications. Triglycerides and Remnant-C levels were log_2_-transformed. ApoA1, apolipoprotein A1; ApoB, apolipoprotein B; BMI, body mass index; BP, blood pressure; HDL-C, high-density lipoprotein cholesterol; LDL-C, low-density lipoprotein cholesterol; LR, likelihood ratio; non-HDL-C, non-high-density lipoprotein cholesterol; Remnant-C, remnant cholesterol; RR, risk ratio; SES, socioeconomic status.

Higher levels of LDL-C/ApoB ratio were inversely associated with MI in both populations, and the RRs (95% CI) were more extreme in the Chinese than in European adults (0.83; 0.77–0.89 vs. 0.92; 0.90–0.95 per 1 SD higher levels, respectively). After further adjustment of triglycerides and ApoA1, the associations of all LDL-related traits with MI remained significant in both populations (see Supplementary material online, *Figure S5*). In addition, multivariable analyses assessing the joint effects of LDL-C and ApoB in CKB demonstrated the independent relevance for MI of ApoB but not of LDL-C (see Supplementary material online, *Table S5*). Indeed, given plasma levels of ApoB, higher plasma LDL-C levels were associated with lower rather than higher risks of MI (see Supplementary material online, *Table S5*). A higher ratio of HDL-C/ApoA1 was associated with higher risk of MI in the Chinese population (RR: 1.30, 95% CI: 1.20–1.41 per SD increase). By contrast, a higher RR (95% CI) of MI for HDL-C/ApoA1 was associated with a lower RR (95% CI) of MI in the European population (0.84; 0.81–0.87 per SD higher levels: *Figure 4*). However, the associations of all HDL-related traits with MI were unaltered after additional adjustment of ApoB and triglycerides in both populations (see Supplementary material online, *Figure S5*). Additional sensitivity analyses assessing the shape and strength of associations of higher levels of triglyceride/HDL-C ratios with MI showed only modest associations with MI that were weaker than those with ApoB or remnant-C in both Chinese and European populations (see Supplementary material online, *Figure S7*). Additional adjustment for chronic kidney disease and antihypertensive medications did not alter the strength of associations of LDL-C, ApoB, triglycerides, or remnant-C with risk of MI in CKB (see Supplementary material online, *Table S6*).

### Sensitivity analyses

Additional sensitivity analyses demonstrated that the strength of associations of LDL-C and ApoB with MI were unaltered by whether the estimates were generated by Cox regression or by logistic regression models in UKB (see Supplementary material online, *Table S7*). In addition, Supplementary material online, *Figure S8* shows that the strength of associations of LDL-C and ApoB with MI in both CKB and UKB populations were unaltered after stratification for elevated vs. normal levels of plasma triglycerides.

## Discussion

This report, involving analyses of contemporary large biobank studies in Chinese and European populations, demonstrated that the mean plasma levels of all LDL-C-related biomarkers were about 30% lower in Chinese than in European populations. Despite the Chinese population having about 30% lower mean levels of all LDL-C-related biomarkers than the European population, the shape and strength of the associations of non-HDL-C and ApoB with risk of MI were remarkably concordant between both populations. The findings might suggest that LDL-C has a greater susceptibility to confounding or reverse causality bias than ApoB. However, the strength of associations of LDL-C with MI was only half as strong in Chinese than in European adults. In contrast, the strength of associations with MI for equivalent proportional differences in ApoB and non-HDL-C were comparable between the Chinese and European adults. The log-linear positive associations of higher levels of non-HDL-C with MI (but not with LDL-C) were consistent with results of a previous study involving a smaller number of MI cases in Chinese adults.^29^

The results for ApoB and non-HDL-C were also concordant with the findings of the Cholesterol Treatment Trialists’ Collaboration (CTTC), which mainly involved European ancestry populations, that demonstrated that the cardiovascular benefits of statin treatment to lower LDL-C levels were independent of baseline LDL-C levels.^30^ The CTTC meta-analysis estimated that a 1 mmol/L lower LDL-C was associated with a 24% lower risk of major coronary events, which is concordant with the findings in the present study of a 24% lower MI risk per 1 mmol/L lower LDL-C in Chinese adults.

Among triglyceride-related traits, equivalent proportional differences in plasma levels of remnant-C were much more strongly associated with MI than triglycerides in both populations. Among HDL-related lipids, the strength of association of ApoA1 with MI was 2-fold greater in CKB than in UKB. Higher levels of LDL particle size were inversely associated with MI in both CKB and UKB, but the pattern for HDL particle size differed between the CKB and UKB. Smaller, denser LDL particles were positively associated with MI in both populations. In contrast, larger HDL particles were positively associated with MI in Chinese but were inversely associated with MI in Europeans (but the reasons for such differences were unexplained).

The stronger associations with MI of remnant-C than triglycerides in both populations are consistent with previous studies in Europeans that demonstrated that remnant-C may be a more important target for residual lipid-related risk beyond LDL-C than triglycerides.^31,32^ Taken together, the findings of the present study highlight the relevance of ApoB measurements beyond LDL-C in Chinese adults when assessing risk of MI. In particular, the findings also suggest that measurements of ApoB and remnant-C levels could inform decision-making on lipid-modifying treatments beyond conventional measures of LDL-C and triglycerides alone for primary prevention of MI when used together with estimation of 10-year absolute risks of ASCVD.^33^

The findings suggest that LDL particles with a lower cholesterol content, representing cholesterol-poor and smaller LDL particles,^3^ may be more strongly associated with MI compared with larger LDL particles. The findings of the present study are consistent with previous studies indicating that, given ApoB, the associations of LDL-C with MI were reversed.^3^ A higher HDL-C to ApoA1 ratio was also associated with higher MI risk in Chinese adults, indicating cholesterol-rich HDL particles, or larger HDL particles, may potentially increase MI risk, which is consistent with a study in European adults.^3^ A prospective study in a Korean population also demonstrated that an increased HDL-C/ApoA1 was associated with a higher risk of MI.^34^ The patterns for HDL-C/ApoA1 observed in Chinese adults differed from those in European adults, possibly reflecting confounding or reverse causality due to alcohol consumption or diabetes, which differed by tertiles of LDL-C/ApoB and HDL-C/ApoA1 ratios in UKB.

The present study also had several limitations. First, despite the prospective study design and exclusion of prior MI or stroke and statin use in CKB, the possibility of residual confounding due to unmeasured confounding factors remains. Second, CKB and UKB are not representative of their respective populations, and the possibility of selection bias cannot be fully excluded, which may limit the generalizability of the findings to the overall Chinese and UK population, respectively. Third, there were marked differences in use of LDL-C-lowering medications between both populations, but the present analyses excluded participants who reported current use of LDL-C-lowering medications at baseline.

While the comparable strength of associations with MI of ApoB and non-HDL-C in CKB provide support for current guidelines for primary prevention of ASCVD that prioritize initiation of statin treatment for individuals with high 10-year absolute risks of ASCVD rather than high levels of LDL-C. The 2019 European Society of Cardiology (ESC) guidelines on statin use recommend use of the Systematic Coronary Risk Evaluation 2 (European-SCORE2) to assess absolute risk of ASCVD with age-specific treatment thresholds (≥7.5% 10-year ASCVD risk if aged 40–49 years and ≥10% if aged 50–69 years).^35^ Different ASCVD risk scores have been suggested for Chinese adults that take account of lower levels of LDL-C and obesity in China.^36^ Local Chinese ASCVD risk scores based on age, systolic and diastolic blood pressures, use of blood pressure–lowering treatment, current daily smoking, self-reported history of diabetes and waist circumference have been advocated, and expert groups also advocate initiation of statins for primary prevention of CVD based on being at high-risk of CVD rather than having high levels of LDL-C.^36,37^

Future research could also include comparative Mendelian randomization (MR) analyses of LDL-C and triglycerides with risk of MI in Chinese and European populations using ancestry-specific genetic variants from GWAS consortia and assess their associations with MI using publicly available GWAS summary statistics of MI in relevant ancestry populations. Such studies would assess the effects of lifelong differences in these lipids on risk of MI in both populations. Likewise, comparative MR analyses in both Chinese and Europeans assessing effects of genetic variants for the key enzymes involved in lipid metabolism on plasma lipid levels could refute ancestry differences in the key enzymes in lipid metabolism. Despite the mean plasma levels of all LDL-C–related biomarkers being 30% lower in Chinese than in European adults, the shape and strength of associations of ApoB and non-HDL-C with risk of MI were concordant between both ancestry populations.

## Lead author biography


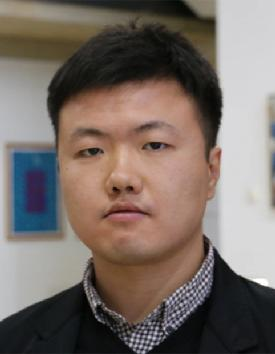


Hanyu Wang, MPhil, is a DPhil student in Population Health at the University of Oxford. His research focuses on plasma lipid traits and cardiovascular disease in diverse populations. He completed his undergraduate studies at Peking University and an MPhil in Epidemiology at the University of Cambridge. He previously worked at the Lee Kong Chian School of Medicine in Singapore on digital health, with research groups in China on chronic disease, and with UNICEF and NGOs on global health. He is interested in using genetic epidemiology to inform strategies for cardiovascular disease prevention.

## Supplementary Material

oeaf119_Supplementary_Data

## Data Availability

Data from baseline, first and second resurveys, and disease follow-up are available under the CKB Open Access Data Policy to bona fide researchers. Sharing of genotyping data is constrained by the Administrative Regulations on Human Genetic Resources of the People's Republic of China. Access to these and certain other data is available through collaboration with CKB researchers. Details of the CKB Data Sharing Policy are available at www.ckbiobank.org. Genotyping data were exported from China to the Oxford CKB International Coordinating Centre under Data Export Approvals 2014–13 and 2015–39 from the Office of Chinese Human Genetic Resource Administration.
