# Supplementary material for: Contrasting associations of blood lipids with risk of myocardial infarction in Chinese and European adults
Source: Eur Heart J Open. 2025 Sep 19;5(5):oeaf119. doi: 10.1093/ehjopen/oeaf119 (PMC12514723; doi:10.1093/ehjopen/oeaf119)
Supplement: oeaf119_Supplementary_Data [file oeaf119_supplementary_data.docx]

**Supplementary Material for**

**Contrasting associations of blood lipids with risk of myocardial infarction in Chinese and European adults**

**Table of contents**

[Members of the China Kadoorie Biobank Collaborative Group 2](#_Toc200968818)

[Table S1. Variable definitions in UKB and CKB 3](#_Toc200968819)

[Table S2. Definition of prevalent and incident CVD outcomes in UKB and CKB 4](#_Toc200968820)

[Table S3. Mean plasma levels of lipids at baseline and resurvey 5](#_Toc200968821)

[Table S4. Prevalence of diabetes, by lipid traits 6](#_Toc200968822)

[Table S5. Multivariable risk ratios (95%CI) of MI after adjustment for additional lipids or apolipoproteins 7](#_Toc200968823)

[Table S6: Risk ratios for additional adjustment for kidney disease on associations of LDL-C, ApoB, triglycerides and remnant-C with MI in CKB 8](#_Toc200968824)

[Table S7. Comparison of logistic and Cox regression results for associations of plasma lipids with risk of MI in UK Biobank 9](#_Toc200968825)

[Figure S1. Flowchart for inclusion of study participants in Chinese and Europeans 10](#_Toc200968826)

[Figure S2. Correlations between different lipid measures in Chinese and Europeans 11](#_Toc200968827)

[Figure S3. Risk ratios (95% CI) of MI by fifths of LDL-C in CKB, excluding individuals with diabetes 12](#_Toc200968828)

[Figure S4. Risk ratios (95% CI) of MI by fifths of LDL-C in UKB, after dividing the first quintile 13](#_Toc200968829)

[Figure S5. Risk ratios (95%CI) of MI before and after additional adjustment for lipids in other classes 14](#_Toc200968830)

[Figure S6. Adjusted RRs (95% CI) of MI for HDL-related measures in Chinese and Europeans 15](#_Toc200968831)

[Figure S7. Risk ratios (95%CI) of MI for ratio of triglycerides to HDL-C in Chinese and Europeans 16](#_Toc200968832)

[Figure S8. Adjusted RRs (95% CI) of MI for LDL-C and ApoB, stratified by plasma TG levels 17](#_Toc200968833)

# Members of the China Kadoorie Biobank Collaborative Group

**International Steering Committee:** Junshi Chen, Zhengming Chen (PI), Robert Clarke, Rory Collins, Liming Li (PI), Jun Lv, Richard Peto, Robin Walters.

**International Co-ordinating Centre, Oxford:** Daniel Avery, Maxim Barnard, Derrick Bennett, Ruth Boxall, Ka Hung Chan, Yiping Chen, Zhengming Chen, Jonathan Clarke, Robert Clarke, Huaidong Du, Ahmed Edris Mohamed, Hannah Fry, Simon Gilbert, Pek Kei Im, Andri Iona, Maria Kakkoura, Christiana Kartsonaki, Kshitij Kolhe, Hubert Lam, Kuang Lin, James Liu, Iona Millwood, Sam Morris, Qunhua Nie, Alfred Pozarickij, Maryam Rahmati, Paul Ryder, Dan Schmidt, Becky Stevens, Iain Turnbull, Robin Walters, Baihan Wang, Lin Wang, Neil Wright, Ling Yang, Xiaoming Yang, Pang Yao.

**National Co-ordinating Centre, Beijing:** Xiao Han, Can Hou, Qingmei Xia, Chao Liu, Jun Lv, Pei Pei, Dianjanyi Sun, Canqing Yu, Lang Pan

**10 Regional Co-ordinating Centres:**

Qingdao CDC: Zengchang Pang, Ruqin Gao, Shanpeng Li, Haiping Duan, Shaojie Wang, Yongmei Liu, Ranran Du, Yajing Zang, Liang Cheng, Xiaocao Tian, Hua Zhang, Yaoming Zhai, Feng Ning, Xiaohui Sun, Feifei Li. Licang CDC: Silu Lv, Junzheng Wang, Wei Hou. Heilongjiang Provincial CDC: Wei Sun, Shichun Yan, Xiaoming Cui. Nangang CDC: Chi Wang, Zhenyuan Wu,Yanjie Li, Quan Kang. Hainan Provincial CDC: Huiming Luo, Tingting Ou. Meilan CDC: Xiangyang Zheng, Zhendong Guo, Shukuan Wu, Yilei Li, Huimei Li. Jiangsu Provincial CDC: Ming Wu, Yonglin Zhou, Jinyi Zhou, Ran Tao, Jie Yang, Jian Su. Suzhou CDC: Fang Liu, Jun Zhang, Yihe Hu, Yan Lu, Liangcai Ma, Aiyu Tang, Shuo Zhang, Jianrong Jin, Jingchao Liu. Guangxi Provincial CDC: Mei Lin, Zhenzhen Lu. Liuzhou CDC: Lifang Zhou, Changping Xie, Jian Lan,Tingping Zhu,Yun Liu, Liuping Wei, Liyuan Zhou, Ningyu Chen, Yulu Qin, Sisi Wang. Sichuan Provincial CDC: Xianping Wu, Ningmei Zhang, Xiaofang Chen, Xiaoyu Chang. Pengzhou CDC: Mingqiang Yuan, Xia Wu, Xiaofang Chen, Wei Jiang, Jiaqiu Liu, Qiang Sun. Gansu Provincial CDC: Faqing Chen, Xiaolan Ren, Caixia Dong. Maiji CDC: Hui Zhang, Enke Mao, Xiaoping Wang, Tao Wang, Xi zhang. Henan Provincial CDC: Kai Kang, Shixian Feng, Huizi Tian, Lei Fan. Huixian CDC: XiaoLin Li, Huarong Sun, Pan He, Xukui Zhang. Zhejiang Provincial CDC: Min Yu, Ruying Hu, Hao Wang. Tongxiang CDC: Xiaoyi Zhang, Yuan Cao, Kaixu Xie, Lingli Chen, Dun Shen. Hunan Provincial CDC: Xiaojun Li, Donghui Jin, Li Yin, Huilin Liu, Zhongxi Fu. Liuyang CDC: Xin Xu, Hao Zhang, Jianwei Chen,Yuan Peng, Libo Zhang, Chan Qu.

# Table S1. Variable definitions in UKB and CKB

| **Variable** | **UKB** | | **CKB** |
| --- | --- | --- | --- |
|  | **Field identifier** | **Definition** | **Definition** |
| ***Sociodemographic*** | | |  |
| Age | 34; 53 | Age when attended assessment centre | Age at baseline |
| Sex | 31 | Sex of participant | Sex of participant |
| Ethnic background | 21000 | Ethnicity from baseline questionnaire | - |
| Townsend deprivation index | 189 | Townsend deprivation index was calculated immediately prior to participant joining UK Biobank | - |
| Educational attainment | 845  6138 | Categories (Field ID: 6138) were mapped onto years of education using the ISCED scale.[4] Those with missing data in categorical educational variables were replaced by age completed full time education (field ID:845) | Self-reported educational attainment, from 0 “No formal school” to 5 “University”. |
| ***Lifestyle factors*** | | |  |
| Alcohol Use | 1558  20117 | Self-reported alcohol use was categorised into 7 categories/  It was further categorized as weekly drinker if the participants were in the "Once or twice a week", "Three or four times a week", "Daily or almost daily" categories. | Self-reported alcohol use with 6 categories, including “weekly” drinkers. |
| Smoking Status | 1239  1249 | Self-reported smoking status was categorised into four categories. "Occasional" and "Regular" smokers were further labelled as current smoker. | Self-reported smoking status was categorised into four categories, including “current smoker”. |
| Physical Activity | 402273 | Sum of MET minutes per week for walking, moderate activity, and vigorous activity, converted to h/day. | MET h/day, including total and work-related physical activity, MET in the analyses was total minus work-related. |
| ***Physical and blood measurement*** | | |  |
| BMI | 21001 | BMI values were constructed from height and weight measured during the initial assessment centre visit. | BMI values were constructed from height and weight at baseline. |
| Systolic Blood Pressure | 4080  93 | Measured twice after participants had sat for at least 5 mins. If automated reading for SBP was not available, manual reading values were used. SBP in the analysis was mean of first and second readings. | Measured twice after participants had sat for at least 5 mins. A third reading of the participants’ SBP was taken if there was a difference between the first two readings of more than 10 mmHg. SBP in the analysis was mean of last two readings. |
| Diastolic Blood Pressure | 4079  94 | Same as systolic blood pressure. | Same as systolic blood pressure. |
| Biochemistry Measurements | | |  |
| Total Cholesterol | 30690 | Measured by CHO-POD analysis on a Beckman Coulter AU5800 | Lipids and apolipoproteins (except LDL-cholesterol and HDL-cholesterol) were assayed using Beckman-Coulter AU680 clinical chemistry analysers.  LDL-cholesterol and HDL-cholesterol were assayed using Ngeneous reagents, calibrations and settings |
| LDL Cholesterol | 17518 | Measured by immunoturbidimetric analysis on a Beckman Coulter AU5800 |  |
| HDL Cholesterol | 30760 |  |  |
| Triglycerides | 30870 |  |  |
| Apolipoprotein B | 30640 |  |  |
| Apolipoprotein A1 | 30630 |  |  |
| HbA1c | 30750 | Measured by HPLC analysis on a Bio-Rad VARIANT II Turbo |  |
| ***Medical History and Medication*** | | |  |
| Diabetes | 20002  6177  6153  2443 | 1) Self-report Diabetes diagnosed by doctor (touch screen)  2) Verbal interview: Diabetes was classified as any diabetes (UKB field identifier 1220), type 1 diabetes (1222), type 2 diabetes (1223), diabetic eye disease (1276), diabetic neuropathy/ulcers (1468), and diabetic nephropathy (1607).  3) Self-reported insulin use.  4) screen-detected: HbA1c ≥6.5% (48 mmol/mol) | Self-reported cases and screen-detected cases (a blood glucose level ≥7.0 mmol/L and a fasting time >8 h, a blood glucose level ≥11.1 mmol/L and a fasting time <8 h, or a fasting blood glucose level ≥7.0 mmol/L). |
| Hypertension | 6177  6153  20002  2443  GP linkage | Hypertension was defined as:  Any antihypertensive use as defined by verbal interview, touchscreen, and GP record.  SBP >= 140 or DBP >=90 | Self-reported hypertension, or having SBP≥140 mmHg or DBP≥90 mmHg, or currently taking antihypertensive medications |
| Antihypertensive and lipid-modifying medications | 20003  6177  6153 | Antihypertensive and lipid-modifying medications at baseline were defined using three sources of information: from verbal interview (Field ID: 20003), GP record linkage, and touchscreen questionnaire (Field ID: 6177, 6153). | Self-reported use of antihypertensive and lipid-modifying medications at baseline |

**Abbreviations**: **UKB**: UK Biobank; **CKB**: China Kadoorie Biobank; **LDL**: Low-Density Lipoprotein; **HDL**: High-Density Lipoprotein; **MET**: Metabolic Equivalent of Task; **BMI**: Body Mass Index; **SBP**: Systolic Blood Pressure; **DBP**: Diastolic Blood Pressure; **CHO-POD**: Cholesterol Oxidase-Peroxidase; **HPLC**: High-Performance Liquid Chromatography; **HbA1c**: Hemoglobin A1c; **GP**: General Practitioner.

# Table S2. Definition of prevalent and incident CVD outcomes in UKB and CKB

| **Conditions** | **UKB** | | | | **CKB** |
| --- | --- | --- | --- | --- | --- |
|  | **HES ICD-10**  **Field ID: 41270** | **Verbal Interview**  **non-cancer illness**  **Field ID:**  **20002** | **Verbal Interview**  **Operations**  **Field ID:**  **20004** | **Touchscreen**  **Medical conditions**  **Field ID:**  **6150, 4056**  **3894** | **Electronic healthcare record linkage** |
| Prevalent MI | I21.x, I22.x, I23.x and diagnosis date is earlier than recruitment date | Verbal interview: self-reported MI diagnosis (1075) | Verbal interview: self-reported operations: coronary angioplasty (PCTA) +/- stent (1070), other arterial surgery/revascularisation procedures (1071), coronary artery bypass grafts (1095), carotid artery surgery/endarterectomy (1105), carotid artery angioplasty +/- stent (1109), coronary angiogram (1514) | Age heart attack diagnosed has value (Field ID: 3894)  Self-reported (Field ID: 6150) heart attack diagnosis | Baseline prevalent CVD cases include reported coronary heart disease, stroke, or transient ischemic attack diagnosis |
| Prevalent Stroke | I60.x, I61.x, I63.x, I64.x and diagnosis date is earlier than recruitment date | Verbal interview: self-reported stroke diagnosis (1081, 1082, 1583, 1491, 1086) |  | Age stroke diagnosed has value (Field ID: 4056)  Self-reported (Field ID: 6150) stroke diagnosis. |  |
| Incident MI | I21.x, I22.x, I23.x and diagnosis date is later than recruitment date |  |  |  | I21.x, I22.x, I23.x and diagnosis date is later than recruitment date |

**Abbreviations**. **HES**: Hospital Episode Statistics; **MI**: Acute Myocardial Infarction; **UKB**: UK Biobank; **CKB**: China Kadoorie Biobank; **CVD**: Cardiovascular Diseases

# Table S3. Mean plasma levels of lipids at baseline and resurvey

|  | **China Kadoorie Biobank** | | | **UK Biobank** | | |
| --- | --- | --- | --- | --- | --- | --- |
|  | Baseline | Resurvey | RDR* | Baseline | Resurvey | RDR |
| ***ApoB, g/L*** | | | | | | |
| Baseline fifths |  |  |  |  |  |  |
| I | 0.57 | 0.69 |  | 0.77 | 0.84 |  |
| II | 0.71 | 0.81 |  | 0.93 | 0.98 |  |
| III | 0.82 | 0.89 |  | 1.05 | 1.06 |  |
| IV | 0.94 | 0.96 |  | 1.16 | 1.13 |  |
| V | 1.12 | 1.08 |  | 1.38 | 1.26 |  |
| Difference (V - I) | 0.55 | 0.40 | ***0.72*** | 0.62 | 0.42 | ***0.68*** |
| ***non-HDL-C, mmol/L*** | | | | | | |
| Baseline fifths |  |  |  |  |  |  |
| I | 2.27 | 2.84 |  | 3.09 | 3.42 |  |
| II | 2.85 | 3.20 |  | 3.82 | 4.00 |  |
| III | 3.29 | 3.57 |  | 4.33 | 4.37 |  |
| IV | 3.76 | 3.92 |  | 4.86 | 4.69 |  |
| V | 4.64 | 4.42 |  | 5.83 | 5.24 |  |
| Difference (V - I) | 2.37 | 1.58 | ***0.67*** | 2.73 | 1.81 | ***0.66*** |
| ***LDL-C, mmol/L*** | | | | | | |
| Baseline fifths |  |  |  |  |  |  |
| I | 1.50 | 1.97 |  | 2.66 | 2.97 |  |
| II | 1.94 | 2.20 |  | 3.26 | 3.43 |  |
| III | 2.28 | 2.52 |  | 3.66 | 3.69 |  |
| IV | 2.65 | 2.79 |  | 4.07 | 3.98 |  |
| V | 3.33 | 3.16 |  | 4.84 | 4.41 |  |
| Difference (V - I) | 1.84 | 1.19 | ***0.65*** | 2.17 | 1.44 | ***0.66*** |
| ***Triglycerides, mmol/L*** | | | | | | |
| Baseline fifths |  |  |  |  |  |  |
| I | 0.76 | 1.25 |  | 0.75 | 1.03 |  |
| II | 1.17 | 1.71 |  | 1.08 | 1.31 |  |
| III | 1.55 | 1.82 |  | 1.41 | 1.58 |  |
| IV | 2.14 | 2.18 |  | 1.90 | 1.86 |  |
| V | 3.69 | 2.93 |  | 3.10 | 2.48 |  |
| Difference (V - I) | 2.92 | 1.68 | ***0.58*** | 2.36 | 1.46 | ***0.62*** |
| ***Remnant-C, mmol/L*** | | | | | | |
| Baseline fifths |  |  |  |  |  |  |
| I | 0.58 | 0.79 |  | 0.35 | 0.46 |  |
| II | 0.79 | 0.92 |  | 0.53 | 0.57 |  |
| III | 0.95 | 1.05 |  | 0.65 | 0.65 |  |
| IV | 1.16 | 1.11 |  | 0.80 | 0.71 |  |
| V | 1.64 | 1.43 |  | 1.12 | 0.87 |  |
| Difference (V - I) | 1.06 | 0.63 | ***0.60*** | 0.76 | 0.41 | **0.*53*** |
| ***ApoA1, g/L*** | | | | | | |
| Baseline fifths |  |  |  |  |  |  |
| I | 1.09 | 1.19 |  | 1.22 | 1.33 |  |
| II | 1.23 | 1.28 |  | 1.40 | 1.47 |  |
| III | 1.34 | 1.37 |  | 1.52 | 1.59 |  |
| IV | 1.45 | 1.44 |  | 1.66 | 1.72 |  |
| V | 1.68 | 1.59 |  | 1.94 | 1.91 |  |
| Difference (V - I) | 0.59 | 0.40 | ***0.69*** | 0.73 | 0.58 | ***0.79*** |
| ***HDL-C, mmol/L*** | | | | | | |
| Baseline fifths |  |  |  |  |  |  |
| I | 0.92 | 1.02 |  | 1.02 | 1.13 |  |
| II | 1.09 | 1.13 |  | 1.26 | 1.35 |  |
| III | 1.23 | 1.25 |  | 1.44 | 1.52 |  |
| IV | 1.39 | 1.34 |  | 1.65 | 1.73 |  |
| V | 1.71 | 1.60 |  | 2.04 | 2.04 |  |
| Difference (V - I) | 0.79 | 0.58 | ***0.73*** | 1.02 | 0.90 | ***0.89*** |

**Abbreviations. RDRs**: Regression Dilution Ratios; **LDL-C**: Low-Density Lipoprotein Cholesterol; **HDL-C**: High-Density Lipoprotein Cholesterol; **ApoB**: Apolipoprotein B; **ApoA1**: Apolipoprotein A1; **Remnant-C**: Remnant Cholesterol
*RDRs were calculated by ratios of interquintile ranges of resurvey and baseline (MacMahon-Peto method).

# Table S4. Prevalence of diabetes, by lipid traits

|  | **China Kadoorie Biobank** | | | | **UK Biobank** | | | |
| --- | --- | --- | --- | --- | --- | --- | --- | --- |
|  | Range | N | Diabetes, % | | Range | | N | Diabetes, % |
| ***LDL-C/ApoB, mmol/g*** | | | |  | | | | |
| Baseline fifths | | | | | |  |  |  |
| I | 1.21 – 2.57 | 1,221 | 6.47 | | 1.85 – 3.33 | | 55,998 | 3.64 |
| II | 2.57 – 2.75 | 1,220 | 4.18 | | 3.33 – 3.45 | | 55,998 | 1.67 |
| III | 2.75 – 2.89 | 1,223 | 3.76 | | 3.45 – 3.55 | | 55,998 | 1.13 |
| IV | 2.89 – 3.05 | 1,217 | 4.35 | | 3.55 – 3.67 | | 55,997 | 0.86 |
| V | 3.05 – 3.77 | 1,220 | 5.16 | | 3.67 – 5.37 | | 55,998 | 0.77 |
| ***Remnant-C, mmol/L*** | | | |  | | | | |
| Baseline fifths | | | | | |  |  |  |
| I | 0.17 – 0.70 | 1,226 | 3.10 | | 0.00 – 0.46 | | 56,073 | 1.40 |
| II | 0.70 – 0.86 | 1,226 | 3.18 | | 0.46 – 0.59 | | 56,111 | 1.34 |
| III | 0.86 – 1.03 | 1,209 | 3.64 | | 0.59 – 0.72 | | 55,882 | 1.43 |
| IV | 1.03 – 1.27 | 1,222 | 5.16 | | 0.72 – 0.90 | | 55,933 | 1.73 |
| V | 1.27 – 3.88 | 1,218 | 8.87 | | 0.90 – 4.18 | | 55,990 | 2.17 |
| ***HDL-C/ApoA1, mmol/g*** | | | |  | | | | |
| Baseline fifths | | | | | |  |  |  |
| I | 0.45 – 0.83 | 1,221 | 8.11 | | 0.49 – 0.86 | | 55,998 | 3.71 |
| II | 0.83 – 0.89 | 1,221 | 6.63 | | 0.86 – 0.92 | | 55,999 | 1.77 |
| III | 0.89 – 0.94 | 1,219 | 4.10 | | 0.92 – 0.97 | | 55,996 | 1.18 |
| IV | 0.94 – 1.00 | 1,220 | 3.03 | | 0.97 – 1.03 | | 55,997 | 0.80 |
| V | 1.00 – 1.39 | 1,220 | 2.05 | | 1.03 – 1.44 | | 55,998 | 0.62 |

**Abbreviations. RRs**: Risk Ratios; **LDL-C**: Low-Density Lipoprotein Cholesterol; **HDL-C**: High-Density Lipoprotein Cholesterol; **ApoB**: Apolipoprotein B; **ApoA1**: Apolipoprotein A1; **Remnant-C:** Remnant Cholesterol.

In the Chinese population, the table was restricted to controls (participants without CVD) only.

# Table S5. Multivariable risk ratios (95%CI) of MI after adjustment for additional lipids or apolipoproteins

|  | China Kadoorie Biobank | UK Biobank |
| --- | --- | --- |
| Exposure | RR (95% CI)  per 1 SD higher | RR (95% CI)  per 1 SD higher |
| ApoB \| Adjust for LDL-C | 1.84 (1.60 - 2.12) | 1.46 (1.37 - 1.55) |
| LDL-C \| Adjust for ApoB | 0.73 (0.62 - 0.86) | 1.08 (1.01 - 1.15) |
| ApoA1 \| Adjust for HDL-C | 0.23 (0.20 - 0.27) | 0.89 (0.84 - 0.94) |
| HDL-C \| Adjust for ApoA1 | 2.31 (1.98 - 2.70) | 0.80 (0.76 - 0.84) |

**Abbreviations. RRs**: Risk Ratios; **LDL-C**: Low-Density Lipoprotein Cholesterol; **HDL-C**: High-Density Lipoprotein Cholesterol; **ApoB**: Apolipoprotein B; **ApoA1**: Apolipoprotein A1

Analyses were conducted by Ridge regression. In the Chinese population, RRs were adjusted for age, sex, region, BMI, diabetes, alcohol use, smoking status, SBP, physical activity, educational attainment, antihypertensive medications, mean temperature, and mean temperature-squared; In European populations, RRs adjusted for age, sex, BMI, diabetes, alcohol use, smoking status, SBP, physical activity, Townsend Deprivation Index, years of education, and antihypertensive medications. | refers to further adjustment.

#

| **Exposure** | **LDL-C** | **ApoB** | **Triglycerides** | **Remnant-C** |
| --- | --- | --- | --- | --- |
| **Chinese** |  |  |  |  |
| Original model | 1.26 (1.11 – 1.42) | 1.44 (1.28 – 1.61) | 1.12 (0.97 – 1.29) | 1.77 (1.53 – 2.05) |
| + history of kidney disease | 1.26 (1.11 – 1.43) | 1.46 (1.30 – 1.64) | 1.12 (0.98 – 1.29) | 1.77 (1.54 – 2.04) |
| + antihypertensives | 1.27 (1.12 – 1.44) | 1.46 (1.30 – 1.64) | 1.12 (0.97 – 1.28) | 1.76 (1.52 – 2.02) |
| + history of kidney disease and antihypertensives | 1.27 (1.12 – 1.44) | 1.46 (1.30 – 1.64) | 1.12 (0.97 – 1.28) | 1.76 (1.52 – 2.02) |
| **Europeans** |  |  |  |  |
| Original model | 1.55 (1.49 – 1.61) | 1.56 (1.51 – 1.62) | 1.44 (1.38 – 1.51) | 1.80 (1.70 – 1.91) |
| + history of chronic kidney disease | 1.55 (1.49 – 1.61) | 1.56 (1.51 – 1.62) | 1.44 (1.37 – 1.50) | 1.80 (1.70 – 1.91) |

# Table S6: Risk ratios for additional adjustment for kidney disease on associations of LDL-C, ApoB, triglycerides and remnant-C with MI in CKB

**Abbreviations:** **ApoB**: Apolipoprotein B; **LDL-C**: Low-Density Lipoprotein Cholesterol; **Remnant-C**: Remnant cholesterol.

In the Chinese population, original models adjusted for age, sex, region, BMI, diabetes, alcohol use, smoking status, SBP, physical activity, educational attainment, mean temperature, and mean temperature-squared;

In European populations, original models were stratified by 5-yrs age-at-risk band and sex, and adjusted for BMI, diabetes, alcohol use, smoking status, SBP, physical activity, Townsend Deprivation Index, years of education, and antihypertensive medications.

# Table S7. Comparison of logistic and Cox regression results for associations of plasma lipids with risk of MI in UK Biobank

|  | Cox Regression | Logistic Regression |
| --- | --- | --- |
| Exposure | HR (95% CI)  per 1 SD higher | OR (95% CI)  per 1 SD higher |
| ApoB | 1.56 (1.51 – 1.62) | 1.59 (1.56 – 1.62) |
| non-HDL-C | 1.59 (1.53 – 1.65) | 1.63 (1.60 – 1.66) |
| LDL-C | 1.55 (1.49 – 1.61) | 1.59 (1.56 – 1.62) |
| Triglycerides | 1.44 (1.38 – 1.51) | 1.47 (1.44 – 1.50) |
| Remnant-C | 1.80 (1.70 – 1.91) | 1.88 (1.82 – 1.94) |
| ApoA1 | 0.72 (0.69 – 0.75) | 0.72 (0.70 – 0.73) |
| HDL-C | 0.72 (0.69 – 0.75) | 0.72 (0.70 – 0.73) |

**Abbreviations**: **OR**: Odds Ratio; **HR**: Hazard Ratio; **ApoB**: Apolipoprotein B; **LDL-C**: Low-Density Lipoprotein Cholesterol; **non-HDL-C**: non-High-Density Lipoprotein Cholesterol; **Remnant-C**: Remnant
Cholesterol; **HDL-C**: High-Density Lipoprotein Cholesterol; **ApoA1**: Apolipoprotein A1; **SD**: Standard Deviation.
For Cox regression models, the HRs were stratified by 5-yrs age-at-risk band and sex, and adjusted for BMI, diabetes, alcohol use, smoking status, SBP, physical activity, Townsend Deprivation Index, years of education, and antihypertensive medications.

For logistic regression models, the ORs were adjusted for age, sex, BMI, diabetes, alcohol use, smoking status, SBP, physical activity, Townsend Deprivation Index, years of education, and antihypertensive medications.

Triglycerides and Remnant-C levels were log-transformed.

# Figure S1. Flowchart for inclusion of study participants in Chinese and Europeans

^
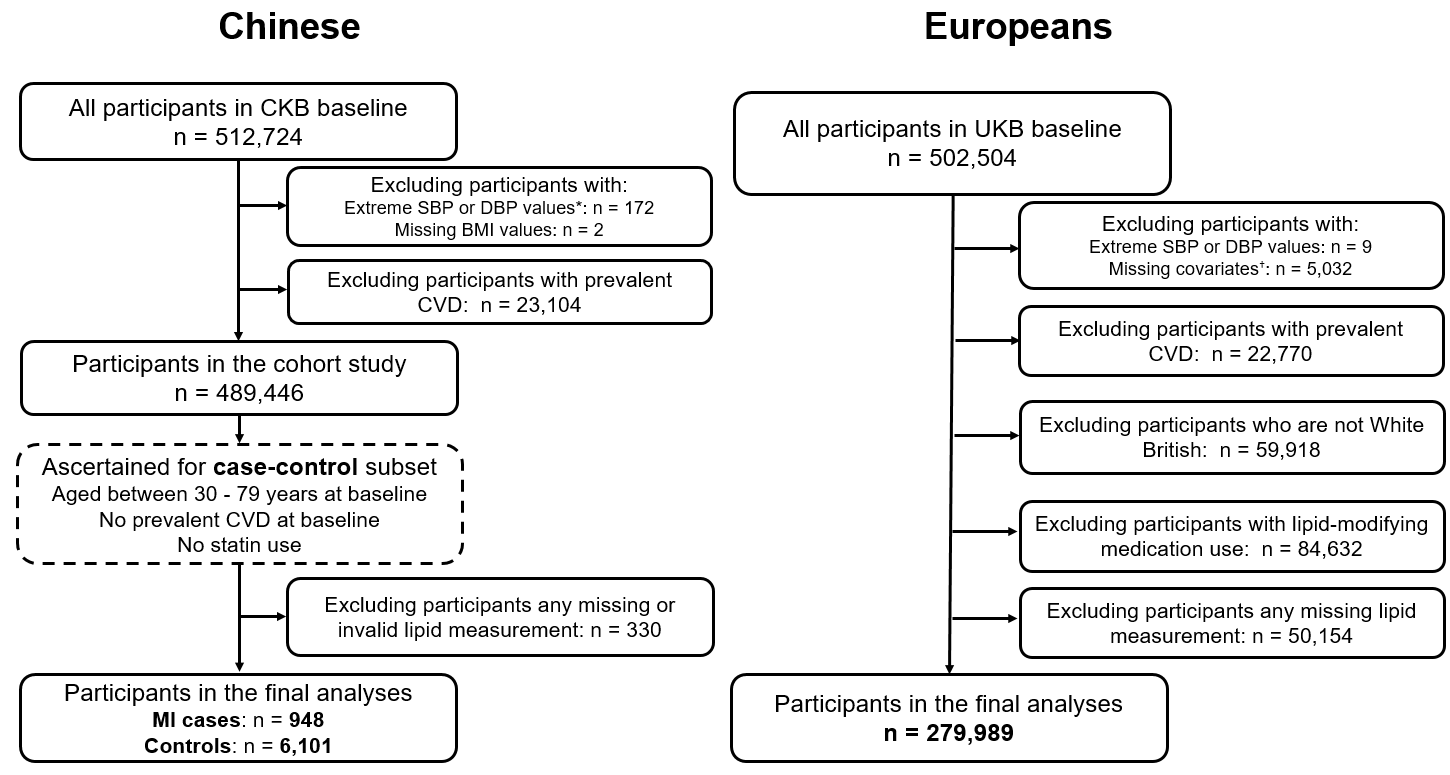
^**Abbreviations**. **CKB**: China Kadoorie Biobank; **SBP**: Systolic Blood Pressure; **DBP**: Diastolic Blood Pressure; **MI**: Myocardial Infarction; **CVD**: Cardiovascular Disease; **BMI**: Body Mass Index
MI cases in the Chinese population were defined at the time of ascertainment.  ^*^Extreme SBP: SBP ≥ 250 or < 80 mmHg and extreme DBP: DBP ≥ 140 or < 40 mmHg ; ^‡^ missing covariates in UKB include: deprivation index (n = 527); BMI (n = 1,937); SBP (n = 797); , educational attainment (n = 1,771)

#
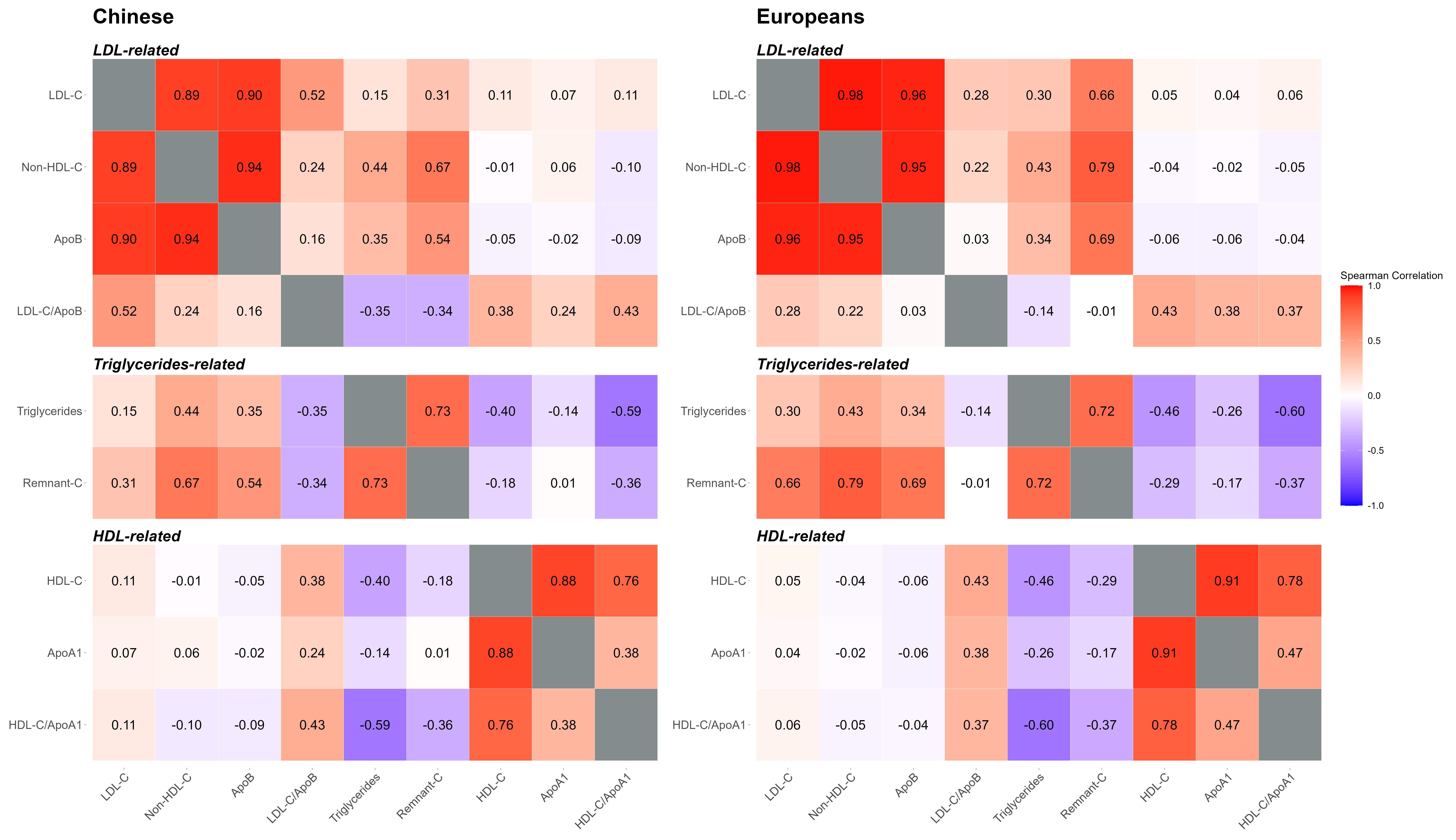
Figure S2. Correlations between different lipid measures in Chinese and Europeans

**Abbreviations**. **ApoB**: Apolipoprotein B; **LDL-C**: Low-Density Lipoprotein Cholesterol; **HDL-C**: High-Density Lipoprotein Cholesterol; **ApoA1**: Apolipoprotein A1; **Remnant-C**: Remnant Cholesterol
Correlations adjusted for age and sex. Correlations in the Chinese population were calculated in controls only.

#
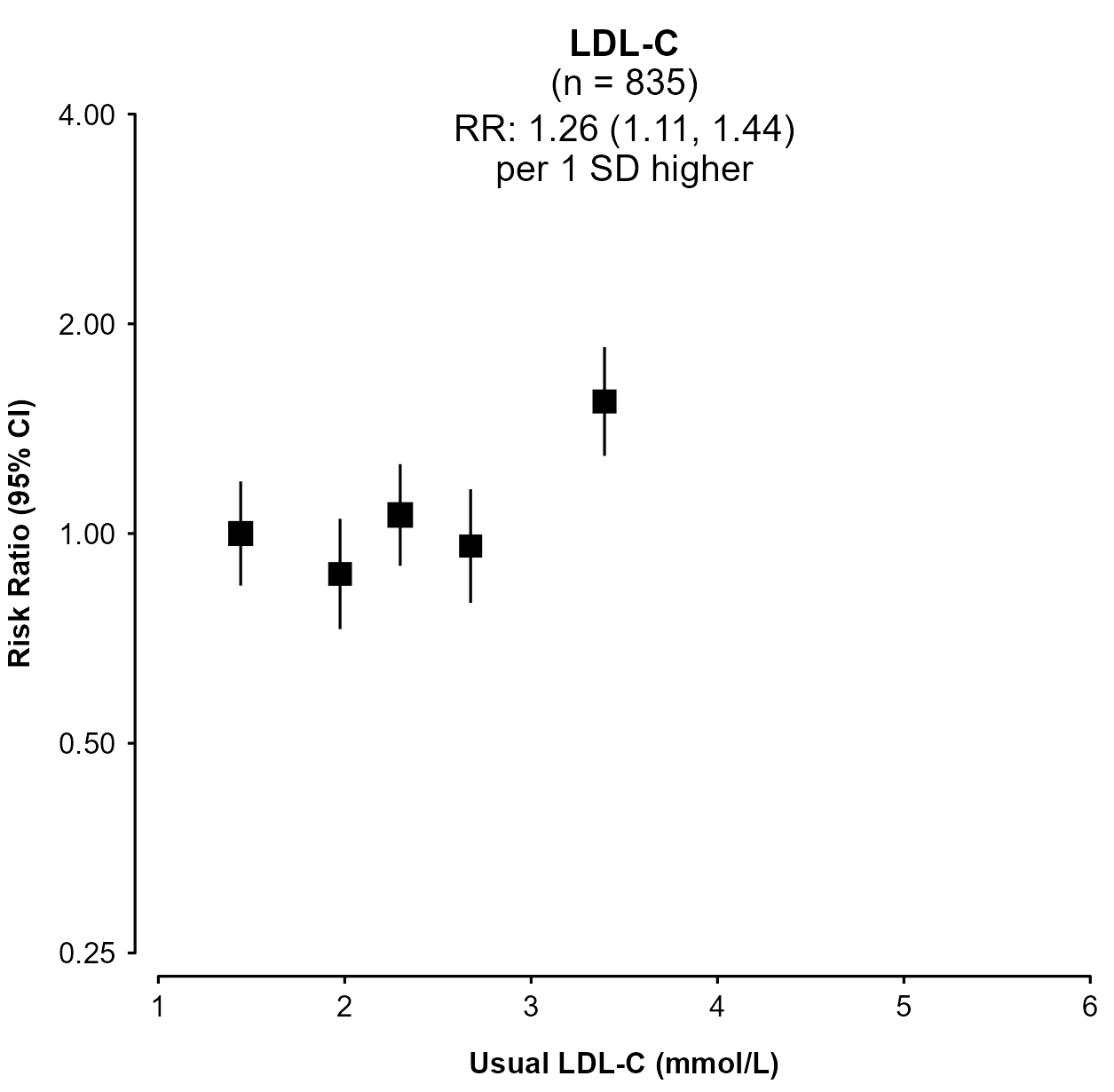
Figure S3. Risk ratios (95% CI) of MI by fifths of LDL-C in CKB, excluding individuals with diabetes

RRs were adjusted for age, sex, region, BMI, alcohol use, smoking status, SBP, physical activity, educational attainment, antihypertensive medications, mean temperature, and mean temperature-squared.

#
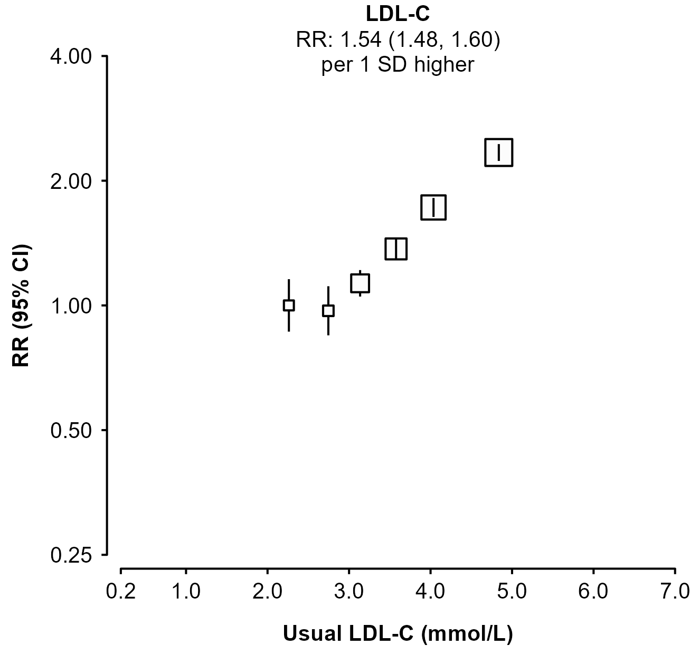
Figure S4. Risk ratios (95% CI) of MI by fifths of LDL-C in UKB, after dividing the first quintile

RRs were stratified by 5-yrs age-at-risk band and sex, and adjusted for BMI, diabetes, alcohol use, smoking status, SBP, physical activity, Townsend Deprivation Index, years of education, and antihypertensive medications.

#
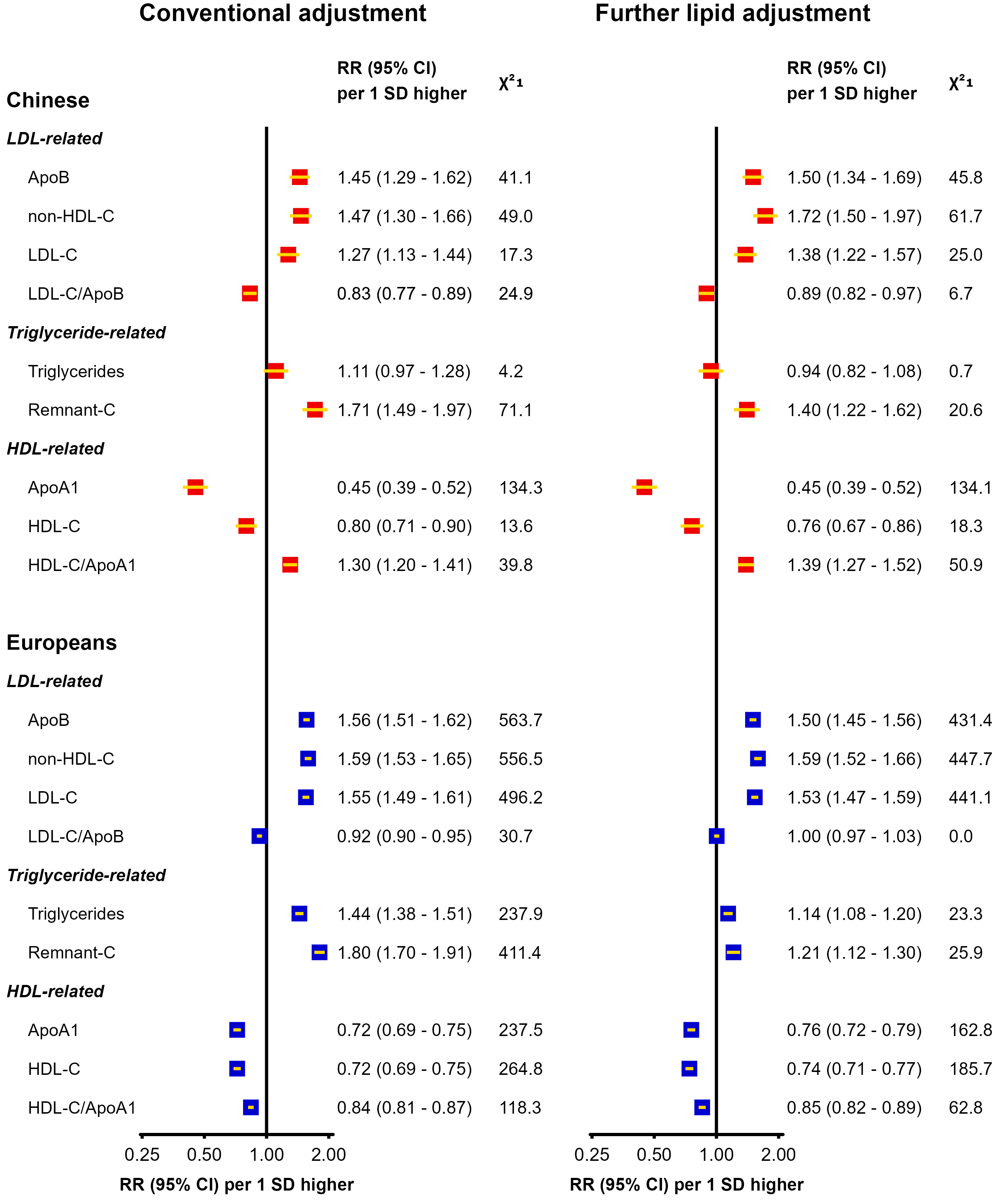
Figure S5. Risk ratios (95%CI) of MI before and after additional adjustment for lipids in other classes

**RRs**: Risk Ratios; **LR**: Likelihood Ratio; **ApoB**: Apolipoprotein B; **LDL-C**: Low-Density Lipoprotein Cholesterol; **non-HDL-C**: non-High-Density Lipoprotein Cholesterol; **Remnant-C**: Remnant Cholesterol**; HDL-C**: High-Density Lipoprotein Cholesterol; **ApoA1**: Apolipoprotein A1; **SD**: Standard Deviation.

In the Chinese population, basic adjustment models adjusted for age, sex, region, BMI, diabetes, alcohol use, smoking status, SBP, physical activity, educational attainment, antihypertensive medications, mean temperature, and mean temperature-squared; In European populations, basic adjusted models were stratified by 5-yrs age-at-risk band and sex, and adjusted for BMI, diabetes, alcohol use, smoking status, SBP, physical activity, Townsend Deprivation Index, years of education, and antihypertensive medications. The χ2 values were computed from likelihood ratio tests with 1 degree of freedom. Triglycerides and Remnant-C levels were log-transformed.
***Further lipid adjustments** include adjusting for lipids in different classes: for LDL-related lipids, adjusting for Triglycerides and ApoA1; for Triglycerides-related lipids, adjusting for ApoB and ApoA1; for HDL-related lipids, adjusting for Triglycerides and ApoB.


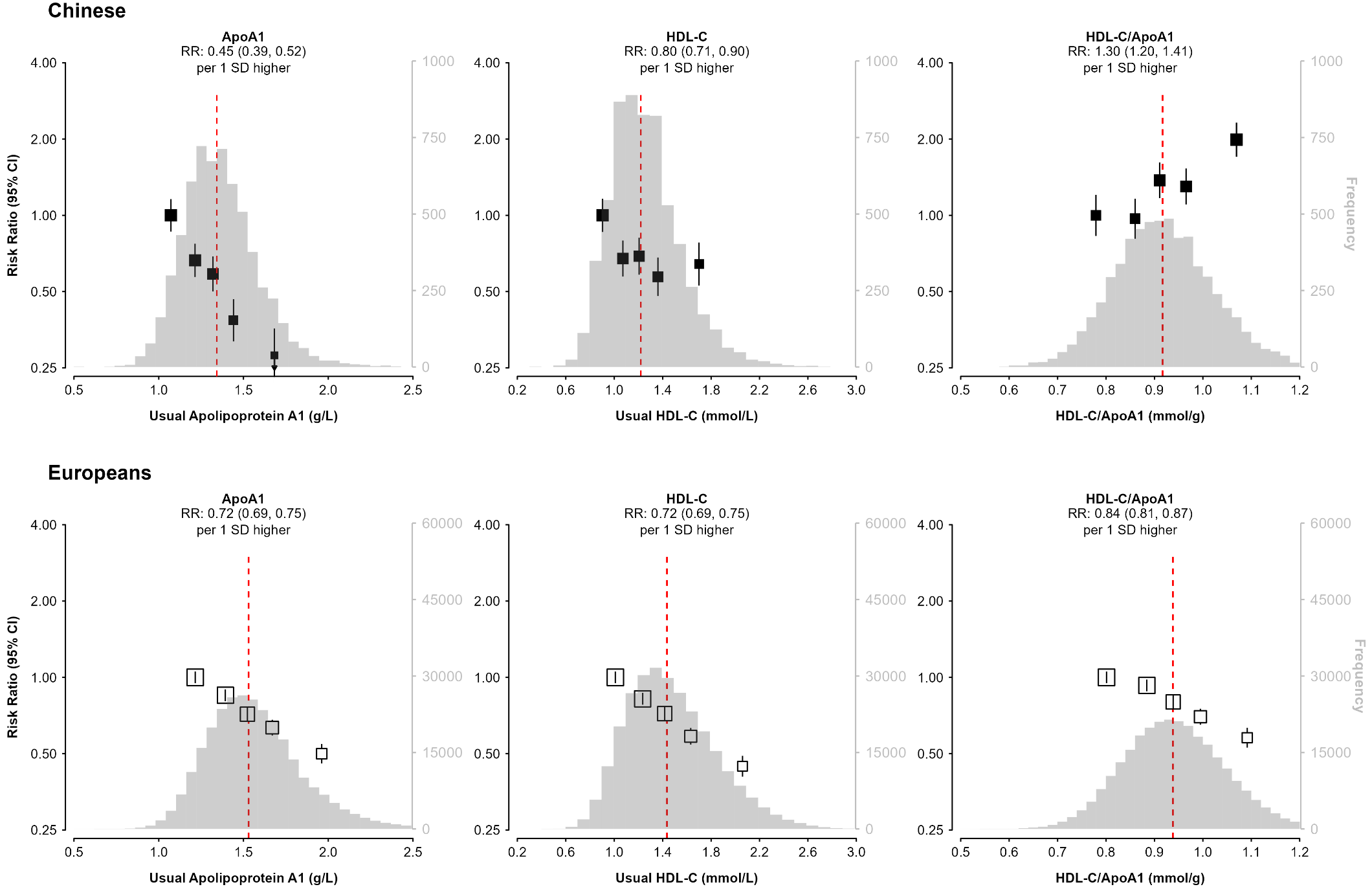
Figure S6. Adjusted RRs (95% CI) of MI for HDL-related measures in Chinese and Europeans
**Abbreviations**: **RRs**: Risk Ratios; **HDL-C:** High-Density Lipoprotein Cholesterol; **ApoA1**: Apolipoprotein A1; **SD**: Standard Deviation.

In CKB, the RRs were adjusted for age, sex, region, BMI, diabetes, alcohol use, smoking status, SBP, physical activity, educational attainment, antihypertensive medications, mean temperature, and mean temperature-squared. In UKB, the RRs were stratified by 5-yrs age-at-risk band and sex, and adjusted for BMI, diabetes, alcohol use, smoking status, SBP, physical activity, Townsend Deprivation Index, years of education, and antihypertensive medications. The distribution of blood lipids were obtained from measurements in baseline samples in controls only. The red dashed lines indicate the median values. SDs are from baseline measurements and can be referred from Table 1.

# Figure S7. Risk ratios (95%CI) of MI for ratio of triglycerides to HDL-C in Chinese and Europeans


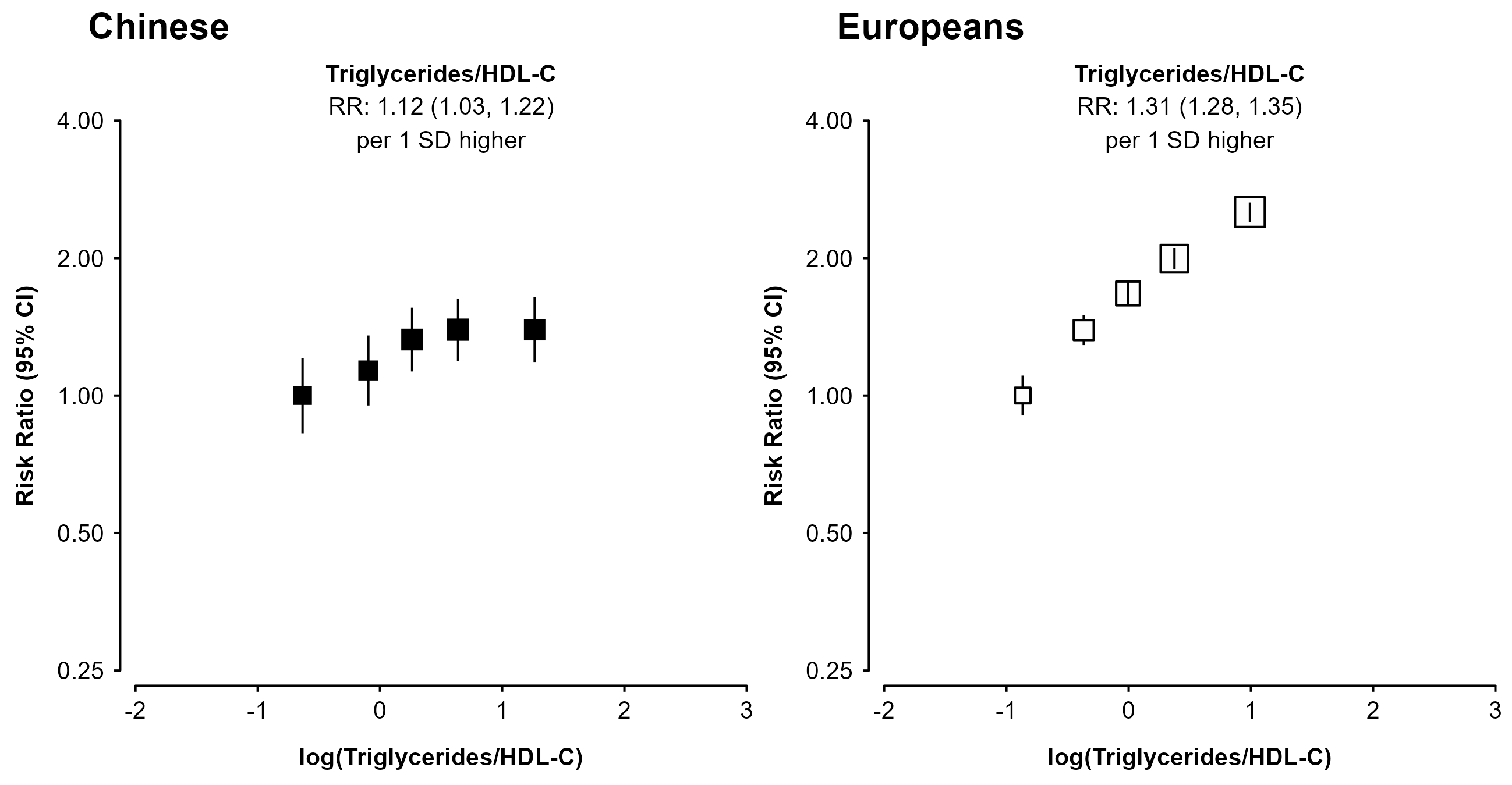


**Abbreviations**. **HDL-C**: High-Density Lipoprotein Cholesterol; **RR**: Risk Ratio; **SD**: Standard Deviation.

In the Chinese population, models adjusted for age, sex, region, BMI, diabetes, alcohol use, smoking status, SBP, physical activity, educational attainment, antihypertensive medications, mean temperature, and mean temperature-squared; In European populations, models were stratified by 5-yrs age-at-risk band and sex, and adjusted for BMI, diabetes, alcohol use, smoking status, SBP, physical activity, Townsend Deprivation Index, years of education, and antihypertensive medications.


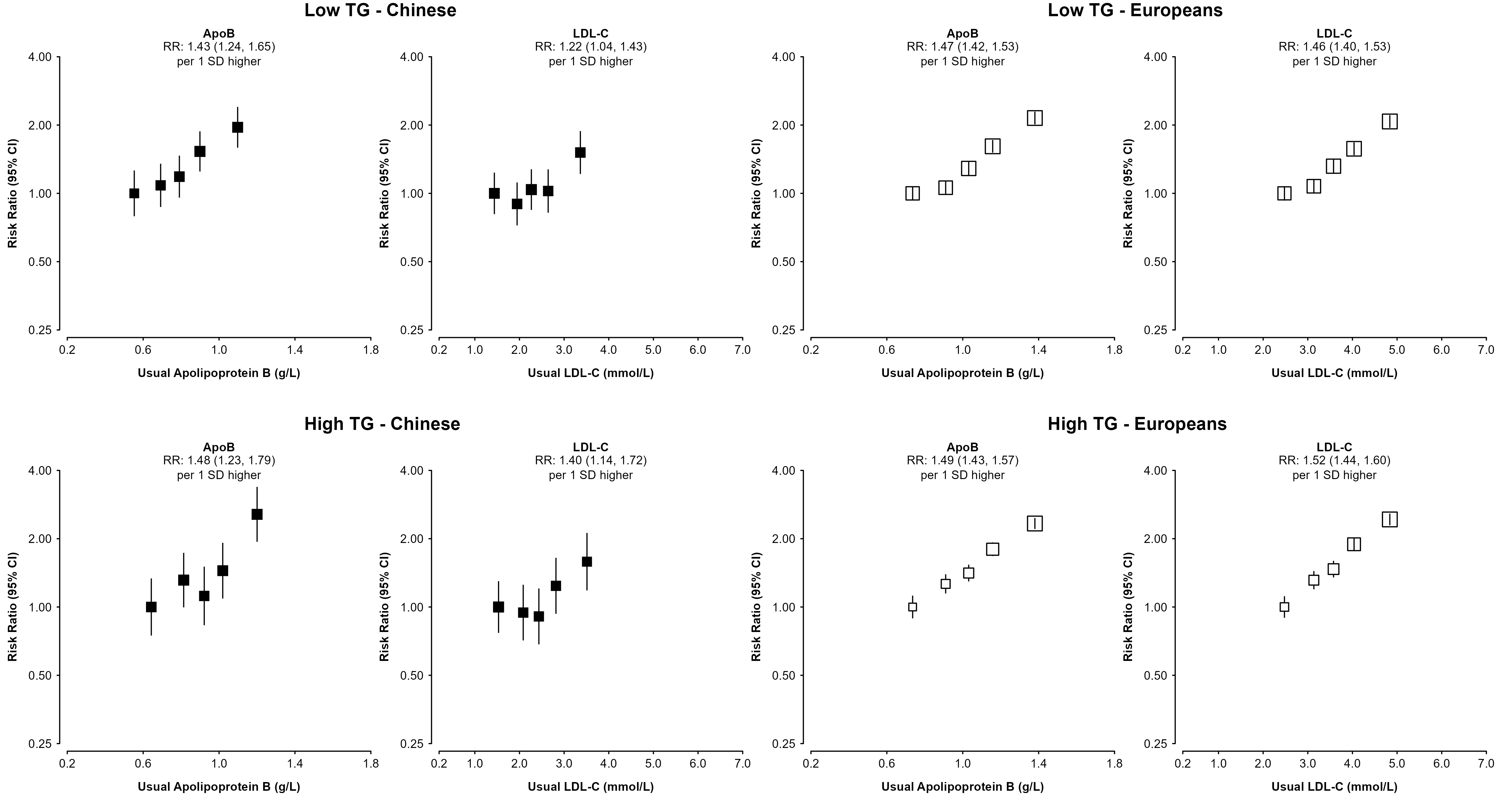
Figure S8. Adjusted RRs (95% CI) of MI for LDL-C and ApoB, stratified by plasma TG levels
**Abbreviations**: **RRs**: Risk Ratios; **ApoB**: Apolipoprotein B; **LDL-C**: Low-Density Lipoprotein Cholesterol; **TG:** Triglycerides; **SD**: Standard Deviation.

Low/High TG are defined as plasma triglycerides levels <2 or ≥ 2 mmol/L

In the Chinese population, the RRs were adjusted for age, sex, region, BMI, diabetes, alcohol use, smoking status, SBP, physical activity, educational attainment, antihypertensive medications, mean temperature, and mean temperature-squared. In European populations, the RRs were stratified by 5-yr age-at-risk band and sex, and adjusted for BMI, diabetes, alcohol use, smoking status, SBP, physical activity, Townsend Deprivation Index, years of education, and antihypertensive medication. The distribution of blood lipids in the Chinese population was measured in baseline samples in controls.
